# Changes in Whole Blood Gene Expression in Obese Subjects with Type 2 Diabetes Following Bariatric Surgery: a Pilot Study

**DOI:** 10.1371/journal.pone.0016729

**Published:** 2011-03-10

**Authors:** Stela Z. Berisha, David Serre, Philip Schauer, Sangeeta R. Kashyap, Jonathan D. Smith

**Affiliations:** 1 Department of Cell Biology, Lerner Research Institute, Cleveland Clinic, Cleveland, Ohio, United States of America; 2 Department of Biological, Geological, and Environmental Sciences, Cleveland State University, Cleveland, Ohio, United States of America; 3 Genomic Medicine Institute, Lerner Research Institute, Cleveland Clinic, Cleveland, Ohio, United States of America; 4 Department of Molecular Medicine, Cleveland Clinic Lerner College of Medicine of Case Western Reserve University, Cleveland, Ohio, United States of America; 5 Bariatric and Metabolic Institute, Cleveland Clinic, Cleveland, Ohio, United States of America; 6 Department of Endocrinology, Diabetes and Metabolism, Cleveland Clinic, Cleveland, Ohio, United States of America; Cardiff University, United Kingdom

## Abstract

**Background:**

A pilot study was performed in order to investigate the effects of bariatric surgery on whole blood gene expression profiles in obese subjects with type 2 diabetes.

**Methodology/Principal Findings:**

Whole blood from eleven obese subjects with type 2 diabetes was collected in PAXgene tubes prior to and 6–12 months after bariatric surgery. Total RNA was isolated, amplified, labeled and hybridized to Illumina gene expression microarrays. Clinical and expression data were analyzed using a paired t-test, and correlations between changes in clinical trait and transcript levels were calculated. Pathways were identified using Ingenuity Pathway Analysis and DAVID gene ontology software. Overall, bariatric surgery resulted in significant reduction of body mass index, fasting plasma glucose, fasting plasma insulin, and normalization of glycosylated hemoglobin levels. The expression levels of 204 transcripts, representing 200 unique genes, were significantly altered after bariatric surgery. Among the significantly regulated genes were *GGT1*, *CAMP*, *DEFA1*, *LCN2*, *TP53*, *PDSS1*, *OLR1*, *CNTNAP5*, *DHCR24*, *HHAT* and *SARDH*, which have been previously implicated in lipid metabolism, obesity and/or type 2 diabetes. Selected findings were replicated by quantitative real-time-PCR. The changes in expression of seven transcripts, *WDR35*, *FLF45244*, *DHCR24*, *TIGD7*, *TOPBP1*, *TSHZ1*, and *FAM8A1* were strongly correlated with the changes in body weight, fasting plasma glucose and glycosylated hemoglobin content. The top pathways associated with gene expression changes after bariatric surgery was lipid metabolism, small molecule biochemistry and gene expression. Two antimicrobial peptides were among the transcripts with the largest changes in gene expression after bariatric surgery.

**Conclusions/Significance:**

Data from this pilot study suggest that whole blood expression levels of specific transcripts may be useful as biomarkers associated with susceptibility for type 2 diabetes and/or therapeutic response.

## Introduction

The prevalence of obesity and type 2 diabetes has increased dramatically in the past decades accompanied by an array of secondary health issues [1]. Both obesity and diabetes are features of the metabolic syndrome, which increases ones risk for cardiovascular diseases. Obesity is a key factor contributing to the development of type 2 diabetes; and, weight loss is associated with improvements in glucose homeostasis. Bariatric surgery represents one of the most efficacious methods for the treatment of obesity and type 2 diabetes; and, it often leads to normalization of hyperglycemia and insulin resistance prior to significant weight loss [2], [3]. Furthermore, bariatric surgery results in sustained weight loss and resolution of type 2 diabetes in a majority of patients with a higher success rate compared to that obtained through diet/lifestyle changes and pharmacotherapy [4].

The main objective of the present pilot study was to examine changes in whole blood gene expression in obese subjects with type 2 diabetes before and after bariatric surgery, which resulted in weight loss and improved hyperglycemia for all subjects. We chose to observe gene expression changes in whole blood, as this tissue may reflect a systemic response to altered metabolism, and it is the easiest tissue to obtain for serial sampling in a clinical setting. In 11 subjects studied before and after surgery, we found 200 unique genes whose expression was significantly altered, many of which have been previously implicated in obesity and/or type 2 diabetes.

## Methods

### Study design

The complete clinical characteristics of subjects that participated in this study are given in Table S1. The study was conducted in accordance with the appropriate clinical and experimental ethical guidelines and was approved by the Cleveland Clinic Institutional Review Board. Written informed consent was obtained by the subjects before their participation. Eleven obese subjects with type 2 diabetes, (5 females and 6 males), with an average age at enrollment 50.5±11.9 (mean ± SD) were studied. Roux-en-Y gastric bypass surgery (RYGB) was performed on seven subjects, (3 females and 4 males), while 4 subjects underwent Sleeve Gastrectomy (SG). None of the women were taking hormonal therapy, no subjects used steroid medications, and none were smokers. Of the 11 subjects, 9 were taking metformin, 2 were taking pioglitazone, and 4 were taking sulfonylureas as oral diabetes medications prior to surgery. Diabetes medications were discontinued by 6 weeks after surgery. Following a 10–12 hour overnight fast, peripheral blood was drawn for clinical laboratory tests and transcriptome analysis 2 weeks prior to and 6–12 months after intervention. All medications were withheld at least 24 hours prior to blood draw on both occasions.

### Isolation of total RNA

Whole blood samples (2.5 ml) from each subject were collected in PAXgene Blood RNA tubes (BD, Franklin Lakes, NJ) which lyses the cells and stabilizes the RNA. Samples were incubated for two hours at room temperature to ensure the complete lysis of blood cells. Total RNA was isolated using PAXgene Blood RNA Kit (Qiagen, Valencia, CA) following manufacturer's guidelines and stored at −80°C. RNA quality was evaluated by incubating 200 ng RNA at 37°C overnight and analyzed by gel electrophoresis.

### Hybridization and microarray data analysis

An aliquot of total RNA for each sample was amplified and biotin labeled using Ovation RNA amplification System V2 (NuGEN, San Carlos, CA). Purified biotin-labeled cDNA was hybridized to HumanWG–6 v2 Expression BeadChip microarrays (Illumina, San Diego, CA) according to the manufacturer's instructions. The hybridization temperature was reduced to 48°C to adjust the conditions for hybridization of cDNA rather than cRNA. The arrays were scanned on the Illumina BeadArray Reader using Illumina BeadScan software. Data were quantile normalized without background subtraction using Illumina BeadStudio software. To reduce the chip-to-chip variability the control (pre-surgery) and post-surgery samples for each patient were put on the same microarray chip. A two-tailed paired t-test analysis was performed to identify the genes whose expression levels changed significantly after the surgery. Only the genes with an unadjusted paired t-test p-value<0.01 were further analyzed and reported. To correct for multiple testing the False Discovery Rate (FDR) Q-value estimates were calculated using QVALUE software (http://www.genomics.princeton.edu/storeylab/qvalue/index.html) [5], [6]. Fold changes in gene expression were calculated as the average of value post/value pre from each of the 11 subjects, and converted to % changes (i.e. 1.40 fold = 40% increase and 0.60 fold = 40% decrease). In order to correlate changes in expression level of each transcript with changes in the clinical values, we calculated, expression levels and clinical values as (value post – value pre)/value pre, and the mean for all subjects was expressed as percent change. Gene ontology, biological networks, and canonical pathways were evaluated with Ingenuity Pathway Analysis software (www.ingenuity.com) and the DAVID Annotation Tool website (http://david.abcc.ncifcrf.gov) [7], [8]. The microarray dataset from this study is available through the Gene Expression Omnibus server, accession number GSE19790. All microarray data are MIAME compliant. Correlation analysis were performed to identify the significantly regulated genes whose changes in expression levels were best associated with changes in weight loss, BMI, fasting plasma glucose, and WBC count.

### Real-time quantitative PCR (qPCR)

To validate the microarray findings, the expression levels of cathelicidin antimicrobial peptide (*CAMP*), alpha defensin 1 (*DEFA1*), and carcinoembrionic antigen-related cell adhesion molecule 8 (*CEACAM8*) were quantified relative to the endogenous control gene, beta actin (*ACTB*) using pre-designed TaqMan gene expression assays (Applied Biosystems, Foster City, CA). Mean fold changes for each sample were calculated by using the 2^−ΔΔCt^ method as previously described [9].

### Protein expression and clinical assays

Human plasma levels of lipocalin 2 (also known as NGAL) were determined using a rapid ELISA kit (BioPorto Diagnostics, Gentofte, Denmark) following the manufacturer's protocol. Serum gamma-glutamyltransferase 1 activity and all other clinical values were determined by standard clinical assays performed by the Cleveland Clinic Pathology and Laboratory Medicine Institute. Gamma-glutamyltransferase activity must be measured in serum, which was only available for six pairs of samples.

## Results

### Clinical and metabolic effects of bariatric surgery

The diabetes related clinical characteristics of the subjects before and after surgery are presented in Table 1 and the complete clinical data for each subject are presented in Table S1. Overall, bariatric surgery had a positive impact on weight loss and resolution of hyperglycemia for every subject included in this study, leading to significant decreases of 22% in mean weight (p = 9.1×10^−7^), 21.0% in mean BMI (p = 9.9×10^−7^), 42% in fasting plasma glucose (p = 8.6×10^−4^), 26% in mean glycosylated hemoglobin (HbA_1C_) content (p = 5.9×10^−4^), and 80% in fasting plasma insulin (p = 0.042). Other significant changes include a 12% decrease in white blood cell count (p = 0.016), a 31% decrease in mean VLDL-cholesterol (p = 0.036), a 54% decrease in plasma alanine aminotransferase activity (p = 7.5×10^−4^), a 7% decrease in % neutrophils (p = 0.01), and a 14% increase in percent lymphocytes (p = 0.007). Although this pilot study was not designed or powered to compare the two types of surgery, we observed that the seven subjects who underwent RYGB surgery were more responsive in weight loss and resolution of type 2 diabetes than the four SG surgery subjects, with larger percent decreases in BMI (24% and 18% decreases for RYGB and SG, respectively, p = 0.016), fasting plasma glucose (45% and 28%, respectively, p = 0.086), and HbA_1C_ (30% and16%, respectively, p = 0.064). Post surgery, all seven of the RYGB subjects and only one of four SG subjects met the criterion for normal fasting plasma glucose of <100 mg/dL (p = 0.007 by chi square test).

**Table 1:** Diabetes related clinical characteristics of subjects involved in the study.

Clinical Characteristic	Pre-surgery^a^	Post-surgery^a^	% Change	P- value^b^	% Change RYGB	%Change SG	P-value^c^
Weight (kg)	140.7±35.1	110.4±29.9	−21.5	9.08E-07	−24	−18	0.033
BMI (kg/m^2^)	47.1±11.6	37.2±10.0	−21.0	9.90E-07	−24	−17	0.016
Fasting plasma glucose (mg/dL)	170±54.2	97.8±26.5	−42.5	8.57E-04	−45	−28	0.086
Fasting plasma insulin (µIU/ml)^d^	32.2±35.4	6.6±4.8	−79.5	4.25E-02	−79	−53	0.135
HbA_1c_ (%)	7.9±1.5	5.9±0.6	−26.3	5.87E-04	−30	−16	0.064

a , Data are means ± SD for 11 subjects pre- and post surgery.

b , Unadjusted paired T-test comparing pre- vs. post surgery.

c , T-test comparing % change by RYBG vs. SG.

d , Data only available for 9 subjects before and after surgery.

BMI, Body mass index.

HbA_1C_, glycosylated hemoglobin A_1C_.

### Gene expression profiling

Of the 48,804 probes on the microarray, 17,115 probe IDs were called present (Illumina detection p-value≤0.05) in 10 or more of the 22 total samples, which were used in subsequent analyses (10 was chosen to reduce the noise while maintaining any potential gender specific expressed transcripts in the five paired female and the six paired male samples). Due to the small number of subjects, we were not powered to compare the effects of the two types of surgery on gene expression, thus all subjects were pooled for our analyses. 204 (∼1.7%) of the transcripts were identified as differentially expressed due to the bariatric surgery at a p-value<0.01 (Table S2). Because some transcripts had more than one probe on the array, these 204 transcripts represent 200 unique genes. To correct for multiple testing, we estimated the false discovery rate (FDR) at p<0.01 to be 0.52, thus, at the considered cutoff we would expect 107 transcripts to appear differentially expressed solely by chance (compared to the 204 differentially expressed transcripts observed). Of the 204 differentially expressed transcripts, 115 (56%) were down regulated and 89 (44%) were up regulated. The top 25 significantly differentially expressed transcripts ranked by p-value are displayed in Table 2. None of the 204 transcripts had a 2- or greater fold change, and only 17 (8.3%) were altered by more than 25%, with 8 being down regulated and 9 up regulated (Table 3).

**Table 2:** Top 25 differentially expressed transcripts and their respective p-values and % expression change after bariatric surgery.

RANK	PROBE_ID	GENE Symbol	Paired T-Test	% Change	DEFINITION
1	ILMN_1652604	GGT1	6.35E-05	−10%	Gamma-glutamyltransferase 1, transcript variant 4.
2	ILMN_1736238	GNMT	9.49E-05	−7%	Glycine N-methyltransferase.
3	ILMN_1688580	CAMP	1.76E-04	−41%	Cathelicidin antimicrobial peptide.
4	ILMN_1682312	CYBB	2.24E-04	−13%	Cytochrome b-245, beta polypeptide (chronic granulomatous disease).
5	ILMN_1768399	ARFIP1	3.64E-04	−9%	ADP-ribosylation factor interacting protein 1, transcript variant 1.
6	ILMN_1781028	ZMYM5	4.18E-04	20%	Zinc finger, MYM-type 5, transcript variant 1.
7	ILMN_1727740	SYNCRIP	4.59E-04	20%	Synaptotagmin binding, cytoplasmic RNA interacting protein.
8	ILMN_1797682	INSL3	4.81E-04	−13%	Insulin-like 3 (Leydig cell).
9	ILMN_1714987	TRIM54	5.04E-04	−18%	Tripartite motif-containing 54, transcript variant 1.
10	ILMN_1675413	ENPP7	5.06E-04	−11%	Ectonucleotide pyrophosphatase/phosphodiesterase 7.
11	ILMN_1813091	ARL1	5.84E-04	9%	ADP-ribosylation factor-like 1.
12	ILMN_1748384	BOC	6.02E-04	15%	Boc homolog (mouse).
13	ILMN_1759017	ZNF333	6.44E-04	15%	Zinc finger protein 333.
14	ILMN_1912287	HS.133181	7.69E-04	−11%	BX093329 Soares_parathyroid_tumor_NbHPA cDNA clone.
15	ILMN_1775570	FLJ44635	8.33E-04	−9%	TPT1-like protein.
16	ILMN_1656371	TRPA1	9.35E-04	−10%	Transient receptor potential cation channel, subfamily A, member 1.
17	ILMN_1689294	LOC85390	9.46E-04	16%	RNA, small nucleolar on chromosome 11.
18	ILMN_1911981	HS.197709	9.90E-04	22%	AGENCOURT_8291102 Lupski_sympathetic_trunk cDNA clone
19	ILMN_1679357	DEFA1/DEFA3	1.08E-03	−46%	Defensin, alpha 1/Defensin alpha 3.
20	ILMN_1804631	CNBP	1.11E-03	13%	CCHC-type zinc finger, nucleic acid binding protein.
21	ILMN_1725661	DEFA1/DEFA3	1.16E-03	−45%	Defensin, alpha 1/Defensin alpha 3.
22	ILMN_1815777	NBPF12	1.16E-03	−11%	PREDICTED: neuroblastoma breakpoint family, member 12.
23	ILMN_1687567	CUTL1	1.25E-03	−10%	Cut-like 1, CCAAT displacement protein, transcript variant 1.
24	ILMN_1672246	OR4C15	1.30E-03	−11%	Olfactory receptor, family 4, subfamily C, member 15.
25	ILMN_1893704	HS.541159	1.31E-03	14%	7n45b12.x1 NCI_CGAP_Lu24 cDNA clone IMAGE:3567335 3.

**Table 3:** Differentially expressed transcripts with >25% change in expression following bariatric surgery.

PROBE_ID	GENE Symbol	P-value^a^	% Change	DEFINITION
ILMN_1679357	DEFA1/DEFA3	1.08E-03	−46%	Defensin, alpha 1/definsin alpha 3.
ILMN_1692223	LCN2	4.37E-03	−46%	Lipocalin 2 (oncogene 24p3).
ILMN_1725661	DEFA1/DEFA3	1.16E-03	−45%	Defensin, alpha 1/defensin alpha 3
ILMN_1693262	DEFA1/DEFA3	1.65E-03	−45%	Defensin, alpha 1/defensin alpha 3
ILMN_1806056	CEACAM8	3.73E-03	−45%	Carcinoembryonic antigen-related cell adhesion molecule 8.
ILMN_1688580	CAMP	1.76E-04	−41%	Cathelicidin antimicrobial peptide.
ILMN_1723035	OLR1	8.09E-03	−37%	Oxidized low density lipoprotein (lectin-like) receptor 1.
ILMN_1762713	C19ORF59	6.53E-03	−29%	Chromosome 19 open reading frame 59.
ILMN_1690546	PPP3CC	6.21E-03	26%	Protein phosphatase 3 catalytic subunit, gamma isoform.
ILMN_1805271	ZNF721	6.81E-03	26%	Zinc finger protein 721.
ILMN_1813400	CBR4	8.96E-03	26%	Carbonyl reductase 4.
ILMN_1702858	ADHFE1	3.78E-03	27%	Alcohol dehydrogenase, iron containing, 1.
ILMN_1748476	NOP5/NOP58	2.49E-03	28%	Nucleolar protein NOP5/NOP58.
ILMN_1661940	CAMTA1	4.40E-03	28%	Calmodulin binding transcription activator 1.
ILMN_1656111	MYLIP	3.83E-03	29%	Myosin regulatory light chain interacting protein.
ILMN_1797893	PFAAP5	5.93E-03	31%	Phosphonoformate immuno-associated protein 5.
ILMN_1679045	SBDS	6.03E-03	39%	Shwachman-Bodian-Diamond syndrome.

a , Paired T-test pre- and post-surgery.

The transcript with the most significant p-value overall encodes for gamma-glutamyltransferase 1 (*GGT1*); and, although its expression was only reduced by 10% post-surgery, this change was consistent in all subjects. The transcript with the third most significant p-value overall encodes for cathelicidin antimicrobial peptide (*CAMP*), which was reduced by 41% post surgery. The microarray contained three probes that align to both alpha defensin 1 (*DEFA1*) and alpha defensin 3 (*DEFA3*), which ranked first, third and fourth of those genes with the largest percent change in gene expression (45 to 46% down regulated, Table 3), demonstrating internal consistency in this finding. Thus, two separate antimicrobial peptides (*CAMP* and *DEFA1/A3*) were relatively highly down regulated. Other transcripts that were relatively highly down regulated include lipocalin 2 (*LCN2*) and carcinoembryonic antigen-related cell adhesion molecule 8 (*CEACAM8*), with 46% and 45% reductions post-surgery, respectively (Table 3).

### Quantitative real-time PCR (qPCR)

To confirm the microarray data we performed qPCR for three relatively highly regulated and robustly expressed transcripts: *DEFA1*, *CAMP*, and *CEACAM8* (Table 4). Paired t-test p-values for the effect of bariatric surgery on transcript expression levels were found to be consistent with the microarray data; and, the percent changes after surgery were similar or greater than the percent changes detected by microarray (Table 4). The expression levels of these three transcripts were compared between the microarray and qPCR data for the 22 (pre and post-surgery) samples using linear regression analysis; and, each transcript had a very strong positive correlation (R values ranged from 0.75 to 0.88).

**Table 4:** qPCR validation of microarray results for select transcripts.

Gene/Transcript Symbol	Array vs. qPCR Correlation (R)^a^	qPCR Paired T-TestP-value^b^	Array Paired T-TestP-value^b^	qPCR% Change	Array% Change
DEFA1	0.88	2.99E-03	1.08E-03	−64%	−46%
CAMP	0.75	1.01E-03	1.76E-04	−43%	−41%
CEACAM8	0.86	1.08E-02	3.73E-03	−57%	−45%

a , P-values of linear regression analysis are all significant (p<0.05).

b , Two-tailed paired t-test based on comparing transcript levels pre- vs. post- surgery.

### Plasma protein assays

To determine whether the protein levels follow the same expression pattern as their transcripts we measured the activity or level of two serum proteins, gamma-glutamyltransferase and lipocalin 2. Serum gamma-glutamyltransferase activity was reduced in the post-surgery samples by an average of 38% (p = 0.05, paired t-test), consistent with the direction of change observed for *GGT1* mRNA levels in whole blood. Plasma levels of lipocalin 2 were determined in 8 subjects pre and post surgery, and its levels were significantly increased after the surgery by an average of 34% (p = 0.008), which is opposite to the whole blood *LCN2* mRNA levels that were significantly decreased. This discrepancy may be due to the majority of plasma lipocalin 2 being synthesized in tissues other than blood such as adipocytes and liver [10], [11].

### Pathway and correlation analyses

**Figure 1:**
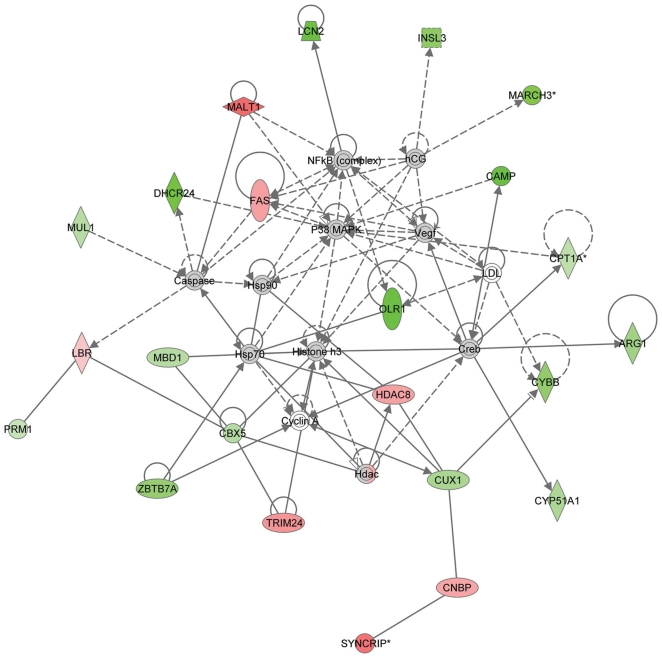
Lipid metabolism, small molecule biochemistry, free radical scavenging network. The straight lines represent direct relationships and the dotted lines represent indirect relationships. Up regulated and down regulated genes that meet the P-value cutoff of <0.01 are shown in red and green shading respectively, with color intensity related to the fold change in expression. The molecules that do not meet the abovementioned P-value cutoff are shown in gray, while the molecules that are incorporated in the network through relationships with other molecules and are not user specified are shown in white. Symbols used in the figure represent: trapezoid, transporter; circle, other; concentric circles, complex/group; dotted square, growth factor; vertical rhombus, enzyme; horizontal rhombus, peptidase; vertical oval, transmembrane receptor; horizontal oval, transcription regulation.

To identify the biological mechanisms, pathways and functions most relevant to the genes of interest, the differentially expressed transcripts (p<0.01) were subjected to Ingenuity Pathway Analysis. The top scoring network identified was “lipid metabolism, small molecule biochemistry and free radical scavenging” (Figure 1). Of the 23 genes involved in the network, 16 were down regulated and seven were up regulated. Also, the differentially expressed transcripts were subjected to canonical pathways analysis via Ingenuity Pathways Analysis software. The following pathways (together with their respective genes) were the most significantly represented canonical pathways (Fisher's exact test p-value<0.05): myc-mediated apoptosis signaling (FAS↑, SHC1↓ and TP53↓); thyroid cancer signaling (SHC1↓ and TP53↓); fatty acid metabolism (ADHFE1↑, CPT1A↓, CYP51A1↓ and SLC27A6↓); iCOS-iCOSL signaling in T-helper cells (PLEKHA3↑, PPP3CC↑ and SHC1↓); and glycine, serine and threonine metabolism (GNMT↓, SARDH↓ and SMOX↓).

Linear regression analysis was performed in order to identify changes in transcript expression that were best correlated with changes in weight, FPG, HbA_1C_, and WBC count, using an arbitrary R^2^ cutoff of ≥0.25. We found that expression changes in 20 regulated transcripts (9.8% of the 204 identified) were highly correlated with changes in weight (Table S3). Of these 20 transcripts, 12 were found to be inversely correlated and 8 were positively correlated with weight change. The transcript best and inversely correlated with changes in weight encodes for prenyl (decaprenyl) diphosphate synthase subunit 1 (*PDSS1*, R = −0.80). Gene ontology (GO) analysis of the 20 transcripts best correlated with weight change revealed two related GO terms that were significantly represented, cholesterol biosynthetic process (p = 1.23E-04) and sterol biosynthetic process (p = 2.21E-04, Table S4).

**Figure 2:**
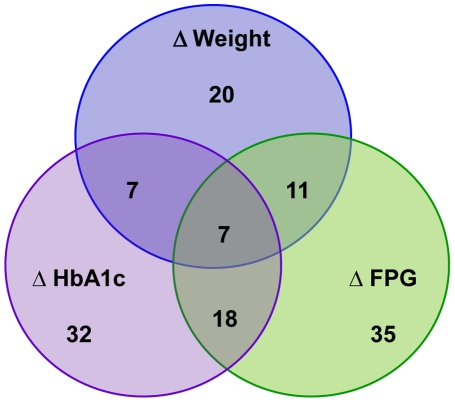
Venn diagram representing the number of transcripts whose change in expression after bariatric surgery were highly correlated with percent changes in weight loss, fasting plasma glucose, and HbA_1C_ content. Seven transcripts were highly correlated with changes in all three of these clinical characteristics.

Changes in 35 regulated transcripts (17.2% of the 204 identified) were highly correlated with changes in FPG. More transcripts were inversely correlated than positively correlated (23 versus 12). The transcript best correlated with changes in FPG was *HHAT* (R = −0.79), which encodes the hedgehog acyltransferase enzyme. Changes in 32 regulated transcripts (15.7% of the 204 identified) were strongly correlated with changes in HbA_1C_ levels, of which 23 were inversely and 9 transcripts were positively correlated (Table S3) The distributions for positive and/or inverse correlations were not significantly different for this or any of the other correlations via chi-square tests. The transcript best and inversely correlated with changes in HbA_1C_ content (R = −0.75) encodes for the WD repeat 35 protein (*WDR35*). We looked for transcripts that were correlated to more than one of these clinical traits, and we found many that were highly correlated with two or more traits, and seven that were highly correlated with all three traits (Figure 2). These seven transcripts were *WDR35*, *FLJ45244*, *DHCR24*, *TIGD7*, *TOPBP1*, *TSHZ1* and *FAM8A1* (Table S3).

Changes in the expression of 27 transcripts (13.24% of the 204 identified) were strongly correlated with changes in WBC count, with 16 inversely and 11 positively correlated. Two of our top three differentially expressed transcripts, *GGT1* and *CAMP*, were highly and positively correlated with changes in WBC count (R = 0.63 and 0.56, respectively, Table S3).

## Discussion

This pilot study was designed to examine the clinical and biochemical changes induced by bariatric surgery and their association with changes in whole blood gene expression in obese subjects with type 2 diabetes. This is the first study that we are aware of comparing the whole blood transcriptome in subjects before and after bariatric surgery. Our pilot study identified ∼200 transcripts whose expression levels in whole blood were significantly changed after bariatric surgery, some of which were previously reported to be implicated in obesity and/or type 2 diabetes. Although we observed consistent reductions in body weight, BMI, fasting plasma glucose, and HbA_1C_ levels following bariatric surgery, we did not specifically measure changes in body fat composition. Thus, we were not able to determine the degree of correlation between changes in gene expression and changes in body fat composition among the 11 subjects. Overall, we observed more changes in gene expression correlated with changes in the measures of diabetes related traits (fasting plasma glucose and HbA_1C_ levels) than we observed with changes in body weight, thus implying that the resolution of diabetes had a stronger influence on whole blood gene expression than the loss of body weight.

We searched the literature and the Gene Expression Omnibus database (http://www.ncbi.nlm.nih.gov/geo) for prior transcriptome profiling studies in humans following bariatric surgery or weight loss. One study examined the skeletal muscle transcriptome in three human subjects before and after bariatric surgery. Twenty genes were differentially expressed using a paired t-test, and all of them were reduced 12 months after surgery with decreases ranging from 25 to 66% [12]. In another study, Ghosh *et al.*, compared whole blood gene expression profiles in lean and obese subjects [13]; although, the Ghosh study used the same source of RNA as the current study, it did not compare subjects prior to and after weight loss using a paired t-test analysis. However, Ghosh deposited additional whole blood gene expression profiles from 10 obese subjects before and after a 6-week diet period which led to weight loss (GEO accession # GSE18897). We analyzed this dataset using the same paired t-test as algorithm as in the current study, and found 388 probes that were significantly different using the unadjusted p-value threshold of <0.01. The expression levels of 194 genes were reduced after weight loss with decreases ranging from 7 to 63%.

In the present study, we found that bariatric surgery led to significantly decreased levels of serum alanine aminotransferase (ALT) (54% decrease), plasma VLDL-cholesterol, and serum gamma-glutamyltransferase (GGT) activity. Given previously reported association of elevated serum VLDL, ALT and GGT levels with hepatic steatosis in obese and type 2 diabetes subjects, and the positive effects of bariatric surgery to reverse hepatic steatosis and the levels of these markers [14], their decreased levels in our study likely reflect the reduction of hepatic fat and improvement in liver function following bariatric surgery. Other studies report that serum levels of GGT are positively and strongly associated with the increased risk of type 2 diabetes over a period of three years [15], [16], and associated with increased risk for fatal cardiovascular disease (CVD) [17]. We found a decrease in whole blood *GGT1* mRNA levels, the transcript with the most significant p-value in our study. Since the liver is thought to be the major source of serum GGT activity [18], we speculate that the liver and blood levels of *GGT1* mRNA may be coordinately regulated. The human *GGT1* gene has multiple promoters and is alternatively spliced leading to several mRNA isoforms [19]. Several studies have reported a complex regulation of *GGT1* transcription upon exposure to oxidative stress [20]; however, it not known whether liver and blood express the same isoforms of *GGT1* mRNA and the pathways that regulate GGT1 expression in these tissues are not well defined. In conclusion, our observed decrease in whole blood GGT1 mRNA levels along with decreased FPG levels after bariatric surgery in the current study are consistent with these prior studies [15]–[17] that showed increased serum GGT activity in those at most risk for type 2 diabetes.

A variety of acute-phase proteins are increased in subjects with obesity and type 2 diabetes [21]; while, several acute phase reactant proteins have been shown to decline following bariatric surgery [22]. We found that the mRNA levels of two antimicrobial peptides, cathelicidin antimicrobial peptide (*CAMP*) and alpha defensin 1/3 (*DEFA1/DEFA3*), were significantly decreased after bariatric surgery. In two prior studies, plasma levels of alpha defensins were found to be decreased by 55 to 60% in women after bariatric surgery, and their levels were correlated with changes in plasma triglyceride levels and pathology based measures of liver steatosis and inflammation [23], [24]. Thus, our finding of decreased *DEFA1/DEFA3* mRNA levels after bariatric surgery agrees with prior findings from plasma protein level measurement assays.

Lipocalin 2 (*LCN2*) is another gene whose expression was significantly (46%) reduced after surgery. *LCN2* has been classified as an adipokine that increases expression of *PPARγ* and adiponectin in 3T3-L1 adipocytes, and suppresses LPS-induced macrophage cytokine production [25]. *Lcn2* mRNA is increased in the liver and adipose tissue of obese *Lepr^db/db^* vs. wild type mice [26]. Also, lipocalin 2 protein levels are higher in obese vs. lean humans [26]. Furthermore, plasma lipocalin 2 levels are positively correlated with BMI, adiposity, hyperglycemia, and insulin resistance [26]. Lipocalin 2 has also been implicated in innate immunity, as *Lcn2*-deficient mice are more susceptible to bacterial infection [26]. Although blood *LCN2* mRNA was decreased after surgery in our study, we found that plasma lipocalin 2 levels increased after surgery, which may be attributed to increased secretion from another tissue source. Although our observed effects of surgery on plasma lipocalin 2 levels are not concordant with the prior correlations, we are not aware of any other studies measuring plasma lipocalin 2 before and after bariatric surgery.

Tumor protein p53 (*TP53*) is another transcript that we identified that was down regulated after bariatric surgery. A recent study has reported that adipose tissue p53 plays a crucial role in the regulation of insulin resistance [27]. Increased expression of *p53* was found to be associated with an increased production of proinflammatory cytokines that led to insulin resistance, while decreased expression had the opposite effect on proinflamatory cytokines and was associated with improved insulin resistance in mice with type 2 diabetes-like disease [27]. Thus, our finding of decreased whole blood *TP53* mRNA after bariatric surgery, weight loss, and remission of type 2 diabetes is consistent with this prior study.

The lectin-like low density lipoprotein receptor 1 (*OLR1*) gene, also known as *LOX1*, encodes for a protein that can be proteolytically cleaved and released as soluble form in serum. Whole blood *OLR1* mRNA levels were significantly decreased by 37% after bariatric surgery in our study, consistent with a previous study reporting that the serum levels of the sLOX1 protein is higher in type 2 diabetes patients than in controls [28].

Human genome wide association studies have identified many SNPs associated with type 2 diabetes. Four genes with common SNPs that were previously identified to be associated with type 2 diabetes [29]–[31] are found in our list of 204 transcripts whose expression was altered after bariatric surgery. Surgery induced changes in expression of all four of these genes were found to be highly correlated with surgery induced changes in HbA_1C_ content. These four genes are: contactin associated protein-like 5 (*CNTNAP5*, decreased after surgery), 24-dehydrocholesterol reductase (*DHCR24*, decreased), hedgehog acyltransferase (*HHAT*, increased), and sarcosine dehydrogenase (*SARDH*, decreased). The finding that two distinct methods, genetic association with type 2 diabetes and transcriptome profiling after bariatric surgery, yield four identical gene hits highlights a potential for gene environmental interactions playing an important role for these specific genes and their influence on glucose homeostasis.

*DHCR24* was also one of seven genes whose change in expression level after bariatric surgery was positively and highly correlated with changes in the three examined clinical phenotypes: HbA_1C_ content, weight, and FPG. *DHCR24* encodes an enzyme participating in sterol biosynthesis; however, it also binds to p53 and protects it from degradation leading to cellular defense against oxidative stress [32], [33]. *DHCR24* is up regulated in endothelial cells by exposure to HDL, and the HDL mediated lowering of *NF-kB* and *VCAM* expression is dependent upon *DHCR24* expression [34]. The decrease in expression of both *DHCR24* and *TP53* in whole blood after surgery reinforces the concept that surgically induced weight loss and remission of type 2 diabetes may alter WBC p53 pathways; however, the role of *DHCR24* in WBC metabolism and inflammation is not known.

One of the advantages of our study is the use of whole blood rather than isolated peripheral mononuclear cells to avoid gene expression changes due to variable handling times during processing [35], in addition to capturing changes in more cell types. Adipose tissue would be an attractive tissue to analyze in regard to changes in gene expression following bariatric surgery, since a higher proportion of adipose transcripts than whole blood transcripts were found to be correlated with BMI in a population study [36]. However, blood is simpler to obtain, particularly for serial sampling; and, genetic cis effects on gene expression are mostly conserved between adipose tissue and whole blood [36]. Furthermore, white blood cell expression profiling has been shown to be useful for screening of diseases of non-blood tissues, such as colorectal cancer [37]. In prior studies, whole blood transcriptome profiling was used to compare obese and lean subjects, yielding sets of transcripts that were differentially expressed [13], [38]. One potential complication of the use of whole blood for transcriptome analysis is that the abundant globin mRNA can interfere with subsequent analyses. There are several ways to counter this issue, and we chose NuGen Ovation RNA amplification kit to eliminate this problem, as it has been shown to be the most efficient method and yields increased sensitivity [39].

The present study had several limitations. The small sample size led to a high FDR and prevented the analysis of differential transcriptome responses to the RYGB and SG surgeries. The paired t-test analysis with each subjects' pre and post surgery whole blood RNA placed on the same Illumina BeadChip controlled for chip to chip variation; but, this design also precluded the use of multiple logistic regression to correct for covariates such as gender and surgery type. Overall, we did not observe large fold effects on transcript levels, but variations of 10% to 60% can have meaningful physiological consequences, particularly in our experimental design that determines changes in whole blood gene expression levels before and after bariatric surgery within each individual subject, thus removing all other genetic and environmental differences among the subjects. The expression of many genes decreased after bariatric surgery, and we presented these reductions as % changes derived from fold changes of post/pre values, which appears to under represent the fold effect. For example, a 50% reduction in gene expression actually represents a −2-fold change. We directly compared the % changes of two transcripts found significantly altered by weight loss in both our study and our analysis of the data from Ghosh *et al.*, (GEO Accession # GSE18897). We observed that expression of *CEACAM8* decreased by 45% (−1.8-fold effect) after bariatric surgery in our study and by 19% (−1.23-fold effect) after diet induced weight loss in the data of Ghosh *et al*. We observed that the expression of *MYLIP* (myosin regulatory light chain interacting protein) increased by 29% (1.29-fold) in our study and by 75% (1.75-fold) in the Ghosh study. Overall, the range of gene expression changes that were decreased after weight loss was fairly comparable in the present study (4 to 46% decreased) to our analysis of the Ghosh data (7 to 63% decreased).

Several of the genes we described whose expression in whole blood changed after bariatric surgery have been previously found to be associated with obesity and/or type 2 diabetes. Thus, one might be able to compare how different diets, drug therapies, and surgical interventions alter whole blood gene expression profiles, and determine the correlations of gene expression changes with clinical improvements in order to examine which pathways are affected by these different therapeutic regimens. In addition baseline whole blood transcriptomes can be examined among subjects who respond differently to different therapeutic treatments for obesity and type 2 diabetes. In the future, whole blood gene expression profiling might be used for personalized medicine in order to make informed clinical decisions among the different therapeutic options.

In conclusion, our pilot study identified many genes whose expression was markedly altered in whole blood following bariatric surgery, some of which are related to inflammation and lipid metabolism. We identified seven genes whose expression changes were correlated with changes in HbA_1C_ content, weight, and FPG. Thus, whole blood expression levels of these and potentially other transcripts identified in this study may be useful as biomarkers indicative of type 2 diabetes susceptibility, as well as response to various therapeutic regimens. In addition, this type of study may illuminate new targets and pathways for intervention.

## Supporting Information

Table S1 **Clinical data measured in 11 subjects that participated in the study before undergoing bariatric surgery (RYGB, Roux-en-Y Gastric Bypass; SG, Sleeve Gastrectomy).** Clinical characteristics that were found to change significantly after bariatric surgery are shown in bold (paired t-test p-value<0.05).
(DOC)

Table S2 **List of significantly differentially expressed (paired t-test p-value<0.01) transcripts IDs with their respective gene symbol, definition and percent change after bariatric surgery.** The genes discussed in the manuscript are shown in bold.
(DOC)

Table S3 **List represents significantly differentially expressed (paired t-test p-value<0.01) transcripts IDs whose changes in gene expression are correlated to changes in at least one of the following four clinical characteristics: weight, FPG, HbA_1C_ or WBC, with an R^2^≥0.25.** Values with R^2^≥0.25 are shown in bold. Gene symbols in blue are correlated to changes in weight, FPG, and HbA_1c_.
(DOC)

Table S4 **Table shows the gene ontology (GO) terms and pathways identified by gene ontology and functional analyses (DAVID) that were overrepresented (P-value<0.01) in our list of 204 significantly differentially expressed transcripts after bariatric surgery.** GO terms discussed in the manuscripts are shown in bold.
(DOC)
