# Supplementary material for: Changes in Whole Blood Gene Expression in Obese Subjects with Type 2 Diabetes Following Bariatric Surgery: a Pilot Study
Source: PLoS One. 2011 Mar 10;6(3):e16729. doi: 10.1371/journal.pone.0016729 (PMC3053356; doi:10.1371/journal.pone.0016729)
Supplement: Table S1 — Clinical data measured in 11 subjects that participated in the study before undergoing bariatric surgery (RYGB, Roux-en-Y Gastric Bypass; SG, Sleeve Gastrectomy). Clinical characteristics that were found to change significantly after bariatric surgery are shown in bold (paired t-test p-value<0.05). (DOC) [file pone.0016729.s001.doc]

**Supplementary Table S1:** Clinical data measured in 11 subjects that participated in the study before undergoing bariatric surgery (RYGB, Roux-en-Y Gastric Bypass; SG, Sleeve Gastrectomy). Clinical characteristics that were found to change significantly after bariatric surgery are shown in bold (paired t-test p-value < 0.05).

|  | **Pre-surgery** | |  |  |  |  |  |  |  |  |  | ***Mean*** | ***SD*** |
| --- | --- | --- | --- | --- | --- | --- | --- | --- | --- | --- | --- | --- | --- |
| *Subject ID* | 1 | 2 | 3 | 4 | 5 | 6 | 7 | 8 | 9 | 10 | 11 |  |  |
| *Type of Surgery* | RYGB | RYGB | RYGB | RYGB | RYGB | RYGB | RYGB | SG | SG | SG | SG |  |  |
| *Age (years)* | 43 | 47 | 50 | 71 | 42 | 44 | 51 | 67 | 61 | 50 | 30 | 50.5 | 11.9 |
| *Gender (F/M)* | M | F | F | M | M | M | F | F | M | F | M | F= 5 | M= 6 |
| *Ht (m)* | 1.80 | 1.65 | 1.72 | 1.77 | 1.79 | 1.76 | 1.73 | 1.55 | 1.83 | 1.66 | 1.77 | 1.7 | 0.1 |
| *Weight (kg)* | 180.5 | 180.2 | 112.7 | 115.8 | 145.5 | 108.0 | 93.2 | 123.5 | 158.0 | 128.9 | 201.3 | 140.7 | 35.1 |
| *Body Mass Index (kg/m^2^)* | 55.71 | 66.19 | 38.01 | 37.05 | 45.41 | 35.06 | 31.14 | 51.40 | 47.23 | 46.78 | 64.25 | 47.1 | 11.6 |
| *Fasting Plasma Glucose (mg/dL)* | 122 | 127 | 205 | 118 | 108 | 220 | 270 | 221 | 176 | 180 | 123 | 170.0 | 54.2 |
| *HbA_1C_* | 6.2 | 6.5 | 8.9 | 6.4 | 8.3 | 10.4 | 10.3 | 7.5 | 8.4 | 7.4 | 7.1 | 7.9 | 1.5 |
| *Fasting Plasma Insulin (µIU/mL)* | 17.3 | - | 17.1 | 13.3 | 32.7 | 24.4 | 24.6 | 8.9 | 124.5 | 27.4 | - | 32.2 | 35.4 |
| *Systolic Blood Pressure (mmHg)* | 110 | 109 | 138 | 142 | 132 | 145 | 137 | 126 | 115 | 101 | 154 | 128.1 | 17.2 |
| *Diastolic Blood Pressure (mmHg)* | 68 | 68 | 97 | 73 | 72 | 91 | 88 | 64 | 73 | 66 | 79 | 76.3 | 11.1 |
| *Total Cholesterol (mg/dL* | 209 | 185 | 189 | 76 | 114 | 283 | 187 | 212 | 152 | 201 | 149 | 177.9 | 54.7 |
| *Triglycerides* | 80 | 384 | 275 | 84 | 105 | 1375 | 171 | 88 | 298 | 124 | 120 | 282.2 | 376.5 |
| *High Density Lipoprotein (mg/dL)* | 28 | 33 | 38 | 27 | 35 | 26 | 44 | 47 | 39 | 41 | 36 | 35.8 | 6.9 |
| *Low Density Lipoprotein (mg/dL)* | 155 | 75 | 96 | 32 | 58 | - | 109 | 147 | 53 | 135 | 89 | 94.9 | 41.7 |
| *Very-Low-Density Lipoprotein (mg/dL)* | 16 | 77 | 55 | 17 | 21 | - | 34 | 18 | 60 | 25 | 24 | 34.7 | 21.6 |
| *Aspartate transaminase (IU/L)* | 22 | 25 | 19 | 29 | 57 | - | - | 34 | 15 | 21 | 26 | 27.6 | 12.4 |
| *Alanine transaminase (U/L)* | 26 | 25 | 29 | 25 | 84 | - | - | 22 | 42 | 31 | 40 | 36.0 | 19.3 |
| *Albumin/creatinine ratio (mg/mmol)* | 17 | 11 | 176 | 5 | 5 | 248 | 49 | 6 | 7 | 62 | 5 | 53.7 | 82.2 |
| *Thyroid Stimulating Hormone (*µ*IU/mL)* | 13.48 | 4.43 | 0.83 | 1.06 | 2.00 | 2.93 | 5.46 | 1.92 | 1.24 | 1.30 | 3.54 | 3.5 | 3.6 |
| *White Blood Cells (x 10^3^/µL)* | 7.22 | 7.70 | 6.52 | 3.98 | 6.60 | 5.00 | 8.46 | 7.19 | 4.78 | 6.06 | 8.68 | 6.6 | 1.5 |
| *Red Blood Cells (x 10^6^/µL)* | 5.03 | 4.32 | 4.92 | 4.30 | 5.13 | 4.55 | 5.04 | 4.51 | 4.41 | 4.59 | 4.70 | 4.7 | 0.3 |
| *Hemoglobin (g/dL)* | 14.3 | 12.7 | 13.4 | 13.2 | 14.4 | 13.6 | 14.1 | 13.5 | 14.0 | 12.9 | 13.5 | 13.6 | 0.6 |
| *Hematocrit (%)* | 42.9 | 38.4 | 41.6 | 37.5 | 43.2 | 39.9 | 42.2 | 41.3 | 42.5 | 39.6 | 41.3 | 40.9 | 1.9 |
| *RDW-CV (%)* | 13.0 | 14.1 | 13.1 | 13.4 | 13.1 | 12.9 | 13.5 | 13.6 | 13.1 | 13.3 | 13.3 | 13.3 | 0.3 |
| *Platelets (x 10^3^/µL)* | 261 | 229 | 314 | 114 | 252 | 191 | 330 | 314 | 209 | 229 | 268 | 246.5 | 62.8 |
| *Percentage of Neutrophils* | 57.2 | 65.2 | 51.1 | 46.0 | 61.2 | - | 46.4 | 77.8 | 58.0 | 66.4 | 61.4 | 59.1 | 9.7 |
| *Absolute Neutrophils* | 4.13 | 5.02 | 3.33 | 1.83 | 4.04 | - | 3.77 | 5.59 | 2.77 | 4.02 | 5.34 | 4.0 | 1.2 |
| *Percentage of Lymphocytes* | 30.2 | 26.0 | 39.6 | 42.2 | 28.3 | - | 45.9 | 16.6 | 29.9 | 25.1 | 28.0 | 31.2 | 8.9 |
| *Absolute Lymphocytes* | 2.18 | 2.00 | 2.58 | 1.68 | 1.87 | - | 3.73 | 1.19 | 1.43 | 1.52 | 2.43 | 2.1 | 0.7 |
| *Percentage of Monocytes* | 7.1 | 6.4 | 5.4 | 8.5 | 7.9 | - | 6.2 | 3.8 | 8.8 | 5.8 | 7.4 | 6.7 | 1.5 |
| *Absolute Monocytes* | 0.51 | 0.49 | 0.35 | 0.34 | 0.52 | - | 0.50 | 0.27 | 0.42 | 0.35 | 0.64 | 0.4 | 0.1 |
| *Percentage of Eosinophils* | 5.1 | 2.1 | 3.1 | 2.8 | 1.8 | - | 1.1 | 1.1 | 2.9 | 2.5 | 2.5 | 2.5 | 1.2 |
| *Absolute Eosinophils* | 0.37 | 0.16 | 0.20 | 0.11 | 0.12 | - | 0.09 | 0.08 | 0.14 | 0.15 | 0.22 | 0.2 | 0.1 |
| *Percentage of Basophils* | 0.4 | 0.3 | 0.8 | 0.5 | 0.8 | - | 0.4 | 0.7 | 0.4 | 0.2 | 0.6 | 0.5 | 0.2 |
| *Absolute Basophils* | 0.03 | 0.02 | 0.05 | 0.02 | 0.05 | - | 0.03 | 0.05 | 0.02 | 0.01 | 0.05 | 0.0 | 0.0 |
| 25-hydroxy vitamin D (ng/mL) | 24 | 25 | 30 | 9 | 29 | 18 | 24 | 19 | 25 | 28 | 16 | 23.1 | 6.3 |

Clinical data measured in 11 subjects that participated in the study 6-12 months after bariatric surgery

|  | **Post - surgery** | | |  |  |  |  |  |  |  |  | ***Mean*** | ***SD*** | ***% Change*** | **Paired T-TEST** |
| --- | --- | --- | --- | --- | --- | --- | --- | --- | --- | --- | --- | --- | --- | --- | --- |
| *Subject ID* | 1 | 2 | 3 | 4 | 5 | 6 | 7 | 8 | 9 | 10 | 11 |  |  |  |  |
| *Type of Surgery* |  |  |  |  |  |  |  |  |  |  |  |  |  |  |  |
| *Age (years)* |  |  |  |  |  |  |  |  |  |  |  |  |  |  |  |
| *Gender (F/M)* |  |  |  |  |  |  |  |  |  |  |  |  |  |  |  |
| *Ht (m)* | 1.80 | 1.65 | 1.70 | 1.78 | 1.81 | 1.75 | 1.72 | 1.52 | 1.82 | 1.66 | 1.77 | 1.7 | 0.1 | -0.3 | 3.20E-01 |
| *Weight (kg)* | 126.2 | 140.6 | 87.5 | 90.9 | 119.2 | 78.0 | 68.8 | 102.1 | 133.4 | 100.1 | 168.0 | 110.4 | 29.9 | -21.5 | **9.08E-07** |
| *Body Mass Index (kg/m^2^)* | 38.95 | 51.64 | 30.28 | 28.69 | 36.38 | 25.62 | 23.26 | 44.19 | 40.27 | 36.33 | 53.62 | 37.2 | 10.0 | -21.0 | **9.90E-07** |
| *Fasting Plasma Glucose (mg/dL)* | 77 | 81 | 88 | 82 | 80 | 77 | 91 | 153 | 139 | 117 | 91 | 97.8 | 26.5 | -42.5 | **8.57E-04** |
| *HbA_1C_* | 5.5 | 5.3 | 5.5 | 4.8 | 5.7 | 6.4 | 5.6 | 7 | 6.4 | 6.3 | 5.9 | 5.9 | 0.6 | -26.3 | **5.87E-04** |
| *Fasting Plasma Insulin (µIU/mL)* | 2.7 | - | 3.7 | 5.6 | 2.5 | 2.4 | 6.7 | 7.6 | 16.6 | 11.7 | - | 6.6 | 4.8 | -79.5 | **4.25E-02** |
| *Systolic Blood Pressure (mmHg)* | 144 | 132 | 144 | 122 | 145 | 149 | 140 | 148 | 138 | 157 | 136 | 141.4 | 9.4 | 10.4 | 7.35E-02 |
| *Diastolic Blood Pressure (mmHg)* | 83 | 72 | 90 | 62 | 82 | 86 | 88 | 69 | 74 | 84 | 97 | 80.6 | 10.3 | 5.7 | 1.79E-01 |
| *Total Cholesterol (mg/dL* | 159 | 169 | 171 | 115 | 101 | 159 | 182 | 177 | 125 | 160 | 178 | 154.2 | 27.7 | -13.3 | 9.69E-02 |
| *Triglycerides* | 88 | 159 | 163 | 79 | 73 | 89 | 119 | 76 | 226 | 84 | 116 | 115.6 | 48.5 | -59.0 | 1.74E-01 |
| *High Density Lipoprotein (mg/dL)* | 43 | 37 | 40 | 35 | 35 | 38 | 67 | 41 | 38 | 39 | 39 | 41.1 | 8.9 | 14.7 | 6.73E-02 |
| *Low Density Lipoprotein (mg/dL)* | 98 | 100 | 98 | 64 | 51 | - | 91 | 121 | 42 | 104 | 116 | 88.5 | 27.0 | -6.7 | 4.97E-01 |
| *Very-Low-Density Lipoprotein (mg/dL)* | 18 | 32 | 33 | 16 | 15 | - | 24 | 15 | 45 | 17 | 23 | 23.8 | 10.0 | -31.4 | **3.59E-02** |
| *Aspartate transaminase (IU/L)* | 20 | 36 | 19 | 25 | 34 | - | - | 14 | 17 | 16 | 11 | 21.3 | 8.7 | -22.6 | 1.29E-01 |
| *Alanine transaminase (U/L)* | 19 | 16 | 11 | 16 | 49 | - | - | 10 | 10 | 10 | 9 | 16.7 | 12.6 | -53.7 | **7.48E-04** |
| *Albumin/creatinine ratio (mg/mmol)* | 7 | 39 | 33 | 6 | 5 | 45 | 8 | 14 | 8 | 43 | - | 20.8 | 17.0 | -61.3 | 1.45E-01 |
| *Thyroid Stimulating Hormone (*µ*IU/mL)* | 34.46 | 2.39 | 0.61 | 1.27 | 1.32 | 6.24 | 2.16 | 1.57 | 1.08 | 1.19 | 2.78 | 5.0 | 9.9 | 44.2 | 4.61E-01 |
| *White Blood Cells (x 10^3^/µL)* | 6.60 | 5.28 | 6.08 | 4.03 | 5.96 | 4.37 | 6.73 | 7.49 | 5.11 | 4.32 | 7.74 | 5.8 | 1.3 | -11.7 | **1.60E-02** |
| *Red Blood Cells (x 10^6^/µL)* | 4.77 | 4.6 | 4.86 | 4.37 | 4.97 | 4.03 | 4.37 | 4.15 | 4.01 | 4.44 | 5.29 | 4.5 | 0.4 | -3.2 | 2.03E-01 |
| *Hemoglobin (g/dL)* | 13.8 | 13.3 | 13.2 | 13.3 | 14.0 | 12.0 | 13.2 | 12.5 | 12.9 | 12.2 | 15.1 | 13.2 | 0.9 | -2.7 | 1.95E-01 |
| *Hematocrit (%)* | 40.6 | 41.9 | 42.0 | 38.6 | 43.4 | 36.0 | 39.8 | 37.8 | 37.7 | 39.4 | 46.9 | 40.4 | 3.1 | -1.4 | 5.67E-01 |
| *RDW-CV (%)* | 14.5 | 13.5 | 15.4 | 13.7 | 13.5 | 12.8 | 12.4 | 13.8 | 12.7 | 13.5 | 13.1 | 13.5 | 0.9 | 1.7 | 4.47E-01 |
| *Platelets (x 10^3^/µL)* | 316 | 206 | 271 | 111 | 224 | 224 | 281 | 260 | 191 | 208 | 235 | 229.7 | 54.2 | -6.8 | 1.31E-01 |
| *Percentage of Neutrophils* | 54.2 | 59.1 | 51.7 | 46.2 | 58.1 | - | 35.9 | 69.8 | 55.0 | 62.8 | - | 54.8 | 9.7 | -7.3 | **9.82E-03** |
| *Absolute Neutrophils* | 3.58 | 3.12 | 3.14 | 1.86 | 3.46 | - | 2.36 | 5.23 | 2.81 | 2.71 | - | 3.1 | 0.9 | -21.2 | **1.68E-02** |
| *Percentage of Lymphocytes* | 31.8 | 28.0 | 39.6 | 42.7 | 32.4 | - | 55.9 | 24.0 | 34.8 | 30.3 | - | 35.5 | 9.5 | 13.9 | **7.07E-03** |
| *Absolute Lymphocytes* | 2.10 | 1.48 | 2.41 | 1.72 | 1.93 | - | 3.68 | 1.80 | 1.78 | 1.31 | - | 2.0 | 0.7 | -1.8 | 9.76E-01 |
| *Percentage of Monocytes* | 8.2 | 8.3 | 6.4 | 6.7 | 6.5 | - | 6.8 | 4.7 | 8.0 | 4.2 | - | 6.6 | 1.5 | -1.3 | 9.81E-01 |
| *Absolute Monocytes* | 0.54 | 0.44 | 0.39 | 0.27 | 0.39 | - | 0.45 | 0.35 | 0.41 | 0.18 | - | 0.4 | 0.1 | -13.4 | 2.12E-01 |
| *Percentage of Eosinophils* | 5.3 | 4.0 | 1.5 | 3.7 | 2.7 | - | 0.8 | 0.8 | 1.8 | 2.5 | - | 2.6 | 1.5 | 2.7 | 8.57E-01 |
| *Absolute Eosinophils* | 0.35 | 0.21 | 0.09 | 0.15 | 0.16 | - | 0.05 | 0.06 | 0.09 | 0.11 | - | 0.1 | 0.1 | -14.0 | 3.66E-01 |
| *Percentage of Basophils* | 0.5 | 0.6 | 0.8 | 0.7 | 0.3 | - | 0.6 | 0.7 | 0.4 | 0.2 | - | 0.5 | 0.2 | 4.6 | 6.74E-01 |
| *Absolute Basophils* | 0.03 | 0.03 | 0.05 | 0.03 | 0.02 | - | 0.04 | 0.05 | 0.02 | 0.01 | - | 0.0 | 0.0 | -5.7 | 1.00E+00 |
| 25-hydroxy vitamin D (ng/mL) | 20 | 22 | 31 | 15 | 31 | 32 | 34 | 21 | 25 | 28 | 19 | 25.3 | 6.3 | 9.4 | 1.52E-01 |
