# Supplementary material for: Changes in Whole Blood Gene Expression in Obese Subjects with Type 2 Diabetes Following Bariatric Surgery: a Pilot Study
Source: PLoS One. 2011 Mar 10;6(3):e16729. doi: 10.1371/journal.pone.0016729 (PMC3053356; doi:10.1371/journal.pone.0016729)
Supplement: Table S2 — List of significantly differentially expressed (paired t-test p-value<0.01) transcripts IDs with their respective gene symbol, definition and percent change after bariatric surgery. The genes discussed in the manuscript are shown in bold. (DOC) [file pone.0016729.s002.doc]

**Supplementary Table S2:** List of significantly differentially expressed (paired t-test p-value < 0.01) transcripts IDs with their respective gene symbol, definition and percent change after bariatric surgery. The genes discussed in the manuscript are shown in bold.

| **P-value RANK** | **PROBE_ID** | **GENE Symbol** | **Paired T-Test** | **% Change** | **DEFINITION** |
| --- | --- | --- | --- | --- | --- |
| 1 | ILMN_1652604 | **GGT1** | 6.35E-05 | -10% | **Gamma-glutamyltransferase 1, transcript variant 4.** |
| 2 | ILMN_1736238 | GNMT | 9.49E-05 | -7% | Glycine N-methyltransferase. |
| 3 | ILMN_1688580 | **CAMP** | 1.76E-04 | -41% | **Cathelicidin antimicrobial peptide.** |
| 4 | ILMN_1682312 | CYBB | 2.24E-04 | -13% | Cytochrome b-245, beta polypeptide (chronic granulomatous disease). |
| 5 | ILMN_1768399 | ARFIP1 | 3.64E-04 | -9% | ADP-ribosylation factor interacting protein 1 (arfaptin 1), transcript variant 1. |
| 6 | ILMN_1781028 | ZMYM5 | 4.18E-04 | 20% | Zinc finger, MYM-type 5, transcript variant 1. |
| 7 | ILMN_1727740 | SYNCRIP | 4.59E-04 | 20% | Synaptotagmin binding, cytoplasmic RNA interacting protein. |
| 8 | ILMN_1797682 | INSL3 | 4.81E-04 | -13% | Insulin-like 3 (Leydig cell). |
| 9 | ILMN_1714987 | TRIM54 | 5.04E-04 | -18% | Tripartite motif-containing 54, transcript variant 1. |
| 10 | ILMN_1675413 | ENPP7 | 5.06E-04 | -11% | Ectonucleotide pyrophosphatase/phosphodiesterase 7. |
| 11 | ILMN_1813091 | ARL1 | 5.84E-04 | 9% | ADP-ribosylation factor-like 1. |
| 12 | ILMN_1748384 | BOC | 6.02E-04 | 15% | Boc homolog (mouse). |
| 13 | ILMN_1759017 | ZNF333 | 6.44E-04 | 15% | Zinc finger protein 333. |
| 14 | ILMN_1912287 | HS.133181 | 7.69E-04 | -11% | BX093329 Soares_parathyroid_tumor_NbHPA cDNA clone IMAGp998A124183 ; IMAGE:1648403. |
| 15 | ILMN_1775570 | FLJ44635 | 8.33E-04 | -9% | TPT1-like protein. |
| 16 | ILMN_1656371 | TRPA1 | 9.35E-04 | -10% | Transient receptor potential cation channel, subfamily A, member 1. |
| 17 | ILMN_1689294 | LOC85390 | 9.46E-04 | 16% | RNA, small nucleolar on chromosome 11. |
| 18 | ILMN_1911981 | HS.197709 | 9.90E-04 | 22% | AGENCOURT_8291102 Lupski_sympathetic_trunk cDNA clone IMAGE:6194146 5. |
| 19 | ILMN_1679357 | **DEFA1/DEFA3** | 1.08E-03 | -46% | **Defensin, alpha 1/Defensin, alpha 3.** |
| 20 | ILMN_1804631 | CNBP | 1.11E-03 | 13% | CCHC-type zinc finger, nucleic acid binding protein. |
| 21 | ILMN_1725661 | **DEFA1/DEFA3** | 1.16E-03 | -45% | **Defensin, alpha 1/Defensin, alpha 3.** |
| 22 | ILMN_1815777 | NBPF12 | 1.16E-03 | -11% | PREDICTED: neuroblastoma breakpoint family, member 12. |
| 23 | ILMN_1687567 | CUTL1 | 1.25E-03 | -10% | Cut-like 1, CCAAT displacement protein (Drosophila), transcript variant 1. |
| 24 | ILMN_1672246 | OR4C15 | 1.30E-03 | -11% | Olfactory receptor, family 4, subfamily C, member 15. |
| 25 | ILMN_1893704 | HS.541159 | 1.31E-03 | 14% | 7n45b12.x1 NCI_CGAP_Lu24 cDNA clone IMAGE:3567335 3. |
| 26 | ILMN_1833316 | HS.112596 | 1.33E-03 | -8% | BX344991 PLACENTA COT 25-NORMALIZED cDNA clone CS0DI054YO01 5-PRIME. |
| 27 | ILMN_1651544 | HDAC8 | 1.37E-03 | 13% | Histone deacetylase 8. |
| 28 | ILMN_1869810 | HS.578254 | 1.43E-03 | -10% | EST98688 Thyroid cDNA 5 end. |
| 29 | ILMN_1896265 | HS.582507 | 1.44E-03 | -10% | CR747973 NCI_CGAP_GC6 cDNA clone IMAGp971B0892 ; IMAGE:2244480 5. |
| 30 | ILMN_1690614 | MYO9A | 1.47E-03 | 8% | Myosin IXA. |
| 31 | ILMN_1751393 | ZNF684 | 1.50E-03 | 11% | Zinc finger protein 684. |
| 32 | ILMN_1890125 | HS.323889 | 1.50E-03 | 13% | yd20d08.s1 Soares fetal liver spleen 1NFLS cDNA clone IMAGE:108783 3. |
| 33 | ILMN_1786319 | MME | 1.54E-03 | -11% | Membrane metallo-endopeptidase, transcript variant 1bis. |
| 34 | ILMN_1772124 | ATRN | 1.56E-03 | 15% | Attractin (ATRN), transcript variant 1. |
| 35 | ILMN_1812701 | C4ORF33 | 1.56E-03 | 24% | Chromosome 4 open reading frame 33, transcript variant 2. |
| 36 | ILMN_1869645 | HS.148758 | 1.61E-03 | -11% | BX095745 Soares_NFL_T_GBC_S1 cDNA clone IMAGp998L204518. |
| **P-value RANK** | **PROBE_ID** | **GENE Symbol** | **Paired T-Test** | **% Change** | **DEFINITION** |
| 37 | ILMN_1898520 | HS.542384 | 1.63E-03 | -10% | xg67h08.x1 NCI_CGAP_Ut4 cDNA clone IMAGE:2633439 3. |
| 38 | ILMN_1693262 | **DEFA1/DEFA3** | 1.65E-03 | -45% | **Defensin, alpha 1/Defensin, alpha 3.** |
| 39 | ILMN_1808132 | FAS | 1.69E-03 | 13% | Fas (TNF receptor superfamily, member 6), transcript variant 3. |
| 40 | ILMN_1703324 | **PDSS1** | 1.73E-03 | -5% | **Prenyl (decaprenyl) diphosphate synthase, subunit 1.** |
| 41 | ILMN_1821269 | HS.541504 | 1.74E-03 | -6% | DB075041 TESTI4 cDNA clone TESTI4018801 5. |
| 42 | ILMN_1769633 | CTSO | 1.96E-03 | 9% | Cathepsin O. |
| 43 | ILMN_1800721 | VPS13A | 1.96E-03 | 11% | Vacuolar protein sorting 13 homolog A (S. cerevisiae), transcript variant D. |
| 44 | ILMN_1802867 | RNASE3 | 1.97E-03 | -21% | Ribonuclease, RNase A family, 3 (eosinophil cationic protein). |
| 45 | ILMN_1654542 | C5ORF21 | 2.00E-03 | 9% | Chromosome 5 open reading frame 21. |
| 46 | ILMN_1904282 | HS.484967 | 2.02E-03 | 10% | Full-length cDNA clone CS0DI042YD07 of Placenta Cot 25-normalized (human) |
| 47 | ILMN_1704973 | **SARDH** | 2.03E-03 | -7% | **Sarcosine dehydrogenase.** |
| 48 | ILMN_1877317 | HS.561625 | 2.13E-03 | 12% | UI-H-BW0-aim-a-09-0-UI.s1 NCI_CGAP_Sub6 cDNA clone IMAGE:2729752 3. |
| 49 | ILMN_1652407 | ZMYND8 | 2.13E-03 | 16% | Zinc finger, MYND-type containing 8, transcript variant 1. |
| 50 | ILMN_1788363 | MLH1 | 2.17E-03 | 15% | MutL homolog 1, colon cancer, nonpolyposis type 2 (E. coli). |
| 51 | ILMN_1900247 | HS.147149 | 2.21E-03 | -7% | BX118294 Soares_testis_NHT cDNA clone IMAGp998H024404. |
| 52 | ILMN_1655796 | MARCH3 | 2.25E-03 | -15% | PREDICTED: membrane-associated ring finger (C3HC4) 3. |
| 53 | ILMN_1651664 | SDHALP1 | 2.26E-03 | -8% | Succinate dehydrogenase complex, subunit A, flavoprotein pseudogene 1 on chromosome 3. |
| 54 | ILMN_1693055 | ZDHHC3 | 2.32E-03 | -13% | Zinc finger, DHHC-type containing 3. |
| 55 | ILMN_1720732 | ZMAT4 | 2.35E-03 | -10% | Zinc finger, matrin type 4. |
| 56 | ILMN_1847128 | HS.564497 | 2.38E-03 | -8% | im45h10.x1 HR85 islet cDNA clone IMAGE:6038251 3. |
| 57 | ILMN_1703858 | C7ORF24 | 2.45E-03 | 11% | Chromosome 7 open reading frame 24. |
| 58 | ILMN_1768743 | FIP1L1 | 2.48E-03 | 9% | FIP1 like 1 (S. cerevisiae). |
| 59 | ILMN_1748476 | NOP5/NOP58 | 2.49E-03 | 28% | Nucleolar protein NOP5/NOP58. |
| 60 | ILMN_1693998 | SNX1 | 2.49E-03 | 24% | Sorting nexin 1, transcript variant 3. |
| 61 | ILMN_1726901 | KLC1 | 2.50E-03 | 15% | Kinesin light chain 1, transcript variant 1. |
| 62 | ILMN_1797576 | PLEKHA3 | 2.54E-03 | 13% | Pleckstrin homology domain containing, family A (phosphoinositide binding specific) member 3. |
| 63 | ILMN_1684346 | TNFAIP8L1 | 2.57E-03 | -11% | Tumor necrosis factor, alpha-induced protein 8-like 1. |
| 64 | ILMN_1667176 | LOC653539 | 2.66E-03 | 13% | PREDICTED: similar to amyotrophic lateral sclerosis 2 (juvenile) chromosome region, candidate 16, transcript variant 5. |
| 65 | ILMN_1681260 | LOC643272 | 2.72E-03 | -15% | PREDICTED: hypothetical protein LOC643272. |
| 66 | ILMN_1754772 | LOC650698 | 2.73E-03 | -7% | PREDICTED: similar to SH3/ankyrin domain gene 2 isoform a. |
| 67 | ILMN_1729175 | FBXO3 | 2.82E-03 | 13% | F-box protein 3, transcript variant 2. |
| 68 | ILMN_1742109 | DNAJC19 | 2.94E-03 | 18% | DnaJ (Hsp40) homolog, subfamily C, member 19. |
| 69 | ILMN_1740217 | HACE1 | 2.95E-03 | -9% | HECT domain and ankyrin repeat containing, E3 ubiquitin protein ligase 1. |
| 70 | ILMN_1687589 | CPT1A | 2.96E-03 | -7% | Carnitine palmitoyltransferase 1A (liver), nuclear gene encoding mitochondrial protein, transcript variant 1. |
| 71 | ILMN_1789535 | DHDDS | 3.02E-03 | 8% | Dehydrodolichyl diphosphate synthase, transcript variant 2. |
| 72 | ILMN_1812578 | LOC645636 | 3.24E-03 | -8% | PREDICTED: similar to AIP1. |
| 73 | ILMN_1666024 | LOC642503 | 3.30E-03 | -9% | PREDICTED: hypothetical protein LOC642503. |
| 74 | ILMN_1726426 | PHF2 | 3.31E-03 | -7% | PHD finger protein 2. |
| 75 | ILMN_1699644 | MARCH3 | 3.33E-03 | -18% | PREDICTED: membrane-associated ring finger (C3HC4) 3. |
| **P-value RANK** | **PROBE_ID** | **GENE Symbol** | **Paired T-Test** | **% Change** | **DEFINITION** |
| 76 | ILMN_1694483 | GPR50 | 3.38E-03 | -9% | G protein-coupled receptor 50. |
| 77 | ILMN_1675543 | PRM1 | 3.40E-03 | -6% | Protamine 1. |
| 78 | ILMN_1661486 | DMRTC1 | 3.47E-03 | -13% | DMRT-like family C1. |
| 79 | ILMN_1821473 | HS.135282 | 3.49E-03 | 19% | cDNA FLJ11554 fis, clone HEMBA1003037 |
| 80 | ILMN_1727526 | KIAA1407 | 3.51E-03 | 14% | KIAA1407. |
| 81 | ILMN_1901720 | HS.540860 | 3.54E-03 | -7% | zw86b02.s1 Soares_total_fetus_Nb2HF8_9w cDNA clone IMAGE:783819 3. |
| 82 | ILMN_1726134 | CBX5 | 3.60E-03 | -8% | Chromobox homolog 5 (HP1 alpha homolog, Drosophila). |
| 83 | ILMN_1672108 | LOC388161 | 3.67E-03 | -6% | PREDICTED: LOC388161, misc RNA. |
| 84 | ILMN_1676905 | **TIGD7** | 3.67E-03 | 19% | **Tigger transposable element derived 7.** |
| 85 | ILMN_1806056 | **CEACAM8** | 3.73E-03 | -45% | **Carcinoembryonic antigen-related cell adhesion molecule 8.** |
| 86 | ILMN_1702858 | ADHFE1 | 3.78E-03 | 27% | Alcohol dehydrogenase, iron containing, 1. |
| 87 | ILMN_1742073 | ADCY1 | 3.82E-03 | -6% | Adenylate cyclase 1 (brain). |
| 88 | ILMN_1656111 | MYLIP | 3.83E-03 | 29% | Myosin regulatory light chain interacting protein. |
| 89 | ILMN_1689378 | CCRN4L | 3.95E-03 | -14% | CCR4 carbon catabolite repression 4-like (S. cerevisiae). |
| 90 | ILMN_1810424 | HRH4 | 3.98E-03 | -14% | Histamine receptor H4. |
| 91 | ILMN_1656118 | ZBTB7A | 4.09E-03 | -12% | Zinc finger and BTB domain containing 7A. |
| 92 | ILMN_1785266 | OFD1 | 4.12E-03 | 19% | Oral-facial-digital syndrome 1. |
| 93 | ILMN_1802185 | CICE | 4.16E-03 | -13% | PREDICTED: cell death-inducing CIDE-like effector pseudogene, transcript variant 2, misc RNA. |
| 94 | ILMN_1814009 | LOC283157 | 4.21E-03 | 8% | PREDICTED: hypothetical LOC283157. |
| 95 | ILMN_1851742 | HS.580430 | 4.22E-03 | -10% | qy47a08.x1 NCI_CGAP_Brn23 cDNA clone IMAGE:2015126 3. |
| 96 | ILMN_1911874 | HS.253554 | 4.28E-03 | -8% | nad32f12.x1 NCI_CGAP_Lu24 cDNA clone IMAGE:3367438 3. |
| 97 | ILMN_1848556 | HS.125695 | 4.33E-03 | -10% | kdef14 Soares_NFL_T_GBC_S1 cDNA clone IMAGE:2349594. |
| 98 | ILMN_1692223 | **LCN2** | 4.37E-03 | -46% | **Lipocalin 2 (oncogene 24p3).** |
| 99 | ILMN_1700518 | HMGN4 | 4.38E-03 | 8% | High mobility group nucleosomal binding domain 4. |
| 100 | ILMN_1661940 | CAMTA1 | 4.40E-03 | 28% | Calmodulin binding transcription activator 1. |
| 101 | ILMN_1815407 | USP16 | 4.49E-03 | 23% | Ubiquitin specific peptidase 16, transcript variant 2. |
| 102 | ILMN_1724634 | LOC645550 | 4.64E-03 | 14% | PREDICTED: hypothetical protein LOC645550. |
| 103 | ILMN_1702783 | LOC652595 | 4.66E-03 | 10% | PREDICTED: similar to U2 small nuclear ribonucleoprotein A (U2 snRNP-A). |
| 104 | ILMN_1660585 | C15ORF40 | 4.67E-03 | 10% | Chromosome 15 open reading frame 40. |
| 105 | ILMN_1804337 | LOC653234 | 4.69E-03 | 9% | PREDICTED: similar to centaurin, gamma-like family, member 1. |
| 106 | ILMN_1844299 | HS.574590 | 4.72E-03 | -17% | DA728582 NT2RM2 cDNA clone NT2RM2002174 5. |
| 107 | ILMN_1820198 | HS.576440 | 4.73E-03 | -13% | DB341807 TESTI4 cDNA clone TESTI4048130 3. |
| 108 | ILMN_1837167 | HS.575696 | 4.76E-03 | -16% | HESC3_16_C05.g1_A036 Human embryonic stem cells cDNA clone IMAGE:7476876 5. |
| 109 | ILMN_1857150 | HS.578899 | 4.79E-03 | 12% | SM016252 Placenta 3 EST cDNA clone ID_16252 3'. |
| 110 | ILMN_1797666 | SLC27A6 | 4.85E-03 | -11% | Solute carrier family 27 (fatty acid transporter), member 6, transcript variant 2. |
| 111 | ILMN_1718354 | INTS7 | 4.97E-03 | 14% | Integrator complex subunit 7. |
| 112 | ILMN_1695719 | EIF2C2 | 4.99E-03 | -16% | Eukaryotic translation initiation factor 2C, 2. |
| 113 | ILMN_1684929 | **TOPBP1** | 5.06E-03 | 16% | **Topoisomerase (DNA) II binding protein 1.** |
| 114 | ILMN_1767000 | SRGAP2 | 5.09E-03 | -8% | SLIT-ROBO Rho GTPase activating protein 2, transcript variant 2. |
| 115 | ILMN_1786564 | LOC497256 | 5.12E-03 | -11% | PREDICTED: hypothetical LOC497256, misc RNA. |
| **P-value RANK** | **PROBE_ID** | **GENE Symbol** | **Paired T-Test** | **% Change** | **DEFINITION** |
| 116 | ILMN_1675055 | C1ORF166 | 5.14E-03 | -8% | Chromosome 1 open reading frame 166. |
| 117 | ILMN_1774971 | **FAM8A1** | 5.29E-03 | 20% | **Family with sequence similarity 8, member A1.** |
| 118 | ILMN_1754976 | LOC647269 | 5.40E-03 | -9% | PREDICTED: similar to Metalloproteinase inhibitor 4 precursor (TIMP-4) (Tissue inhibitor of metalloproteinases-4). |
| 119 | ILMN_1888435 | FAM39DP | 5.47E-03 | -16% | Family with sequence similarity 39, member D pseudogene on chromosome 15. |
| 120 | ILMN_1738246 | OR10A7 | 5.53E-03 | -12% | Olfactory receptor, family 10, subfamily A, member 7. |
| 121 | ILMN_1857743 | HS.569496 | 5.55E-03 | -5% | tt04g12.x1 NCI_CGAP_GC6 cDNA clone IMAGE:2239846 3 similar to contains element MER32 MER32 repetitive element ;. |
| 122 | ILMN_1724240 | LBR | 5.69E-03 | 8% | Lamin B receptor, transcript variant 2. |
| 123 | ILMN_1718907 | **TSHZ1** | 5.73E-03 | 24% | **Teashirt zinc finger homeobox 1.** |
| 124 | ILMN_1910972 | HS.561924 | 5.82E-03 | -14% | UI-E-EJ0-ahg-e-04-0-UI.s1 UI-E-EJ0 cDNA clone UI-E-EJ0-ahg-e-04-0-UI 3. |
| 125 | ILMN_1700307 | FLJ38969 | 5.84E-03 | 19% | PREDICTED: hypothetical protein FLJ38969. |
| 126 | ILMN_1831119 | HS.578507 | 5.91E-03 | -10% | 17000424188250 GRN_ES cDNA 5. |
| 127 | ILMN_1797893 | PFAAP5 | 5.93E-03 | 31% | Phosphonoformate immuno-associated protein 5. |
| 128 | ILMN_1662318 | CCDC59 | 5.99E-03 | 21% | Coiled-coil domain containing 59. |
| 129 | ILMN_1742608 | SMOX | 6.03E-03 | -9% | Spermine oxidase, transcript variant 4. |
| 130 | ILMN_1679045 | SBDS | 6.03E-03 | 39% | Shwachman-Bodian-Diamond syndrome. |
| 131 | ILMN_1654493 | LOC649169 | 6.04E-03 | -4% | PREDICTED: similar to WD-repeat protein 74 (NOP seven-associated protein 1), transcript variant 1. |
| 132 | ILMN_1779356 | **TP53** | 6.10E-03 | -7% | **Tumor protein p53 (Li-Fraumeni syndrome).** |
| 133 | ILMN_1738976 | OR2A20P | 6.13E-03 | 13% | Olfactory receptor, family 2, subfamily A, member 20 pseudogene on chromosome 7. |
| 134 | ILMN_1690546 | PPP3CC | 6.21E-03 | 26% | Protein phosphatase 3 (formerly 2B), catalytic subunit, gamma isoform. |
| 135 | ILMN_1664718 | **CYP51A1** | 6.34E-03 | -9% | **Cytochrome P450, family 51, subfamily A, polypeptide 1.** |
| 136 | ILMN_1730986 | MALT1 | 6.36E-03 | 22% | Mucosa associated lymphoid tissue lymphoma translocation gene 1, transcript variant 1. |
| 137 | ILMN_1689234 | LOC643836 | 6.39E-03 | 13% | PREDICTED: similar to Zinc finger protein 62 homolog (Zfp-62) (ZT3). |
| 138 | ILMN_1693853 | **HHAT** | 6.40E-03 | 14% | **Hedgehog acyltransferase.** |
| 139 | ILMN_1762713 | C19ORF59 | 6.53E-03 | -29% | Chromosome 19 open reading frame 59. |
| 140 | ILMN_1859007 | HS.541685 | 6.56E-03 | -11% | wf40a08.x1 Soares_NFL_T_GBC_S1 cDNA clone IMAGE:2358038 3 similar to contains Alu repetitive element;. |
| 141 | ILMN_1805216 | GPC6 | 6.76E-03 | -9% | Glypican 6. |
| 142 | ILMN_1727674 | FOXD4L2 | 6.77E-03 | -11% | Forkhead box D4-like 2. |
| 143 | ILMN_1805271 | ZNF721 | 6.81E-03 | 26% | Zinc finger protein 721. |
| 144 | ILMN_1652120 | ZNF302 | 6.86E-03 | 9% | Zinc finger protein 302, transcript variant 2. |
| 145 | ILMN_1740258 | KRTAP20-2 | 6.98E-03 | -7% | Keratin associated protein 20-2. |
| 146 | ILMN_1652409 | SPATA7 | 7.06E-03 | 12% | Spermatogenesis associated 7, transcript variant 2. |
| 147 | ILMN_1777449 | IFT74 | 7.13E-03 | 12% | Intraflagellar transport 74 homolog (Chlamydomonas), transcript variant 2. |
| 148 | ILMN_1820761 | HS.106801 | 7.13E-03 | -11% | qi02a09.x1 Soares_NFL_T_GBC_S1 cDNA clone IMAGE:1855288 3. |
| 149 | ILMN_1819255 | HS.428820 | 7.18E-03 | -9% | UI-H-CO0-asj-h-04-0-UI.s1 NCI_CGAP_Sub9 cDNA clone UI-H-CO0-asj-h-04-0-UI 3. |
| 150 | ILMN_1751793 | PCNXL2 | 7.40E-03 | 9% | Pecanex-like 2 (Drosophila), transcript variant 1. |
| 151 | ILMN_1764619 | **FLJ45244** | 7.41E-03 | 11% | **FLJ45244 protein.** |
| 152 | ILMN_1819332 | HS.583211 | 7.43E-03 | -12% | UI-H-CO0-atb-h-04-0-UI.s1 NCI_CGAP_Sub9 cDNA clone UI-H-CO0-atb-h-04-0-UI 3. |
| 153 | ILMN_1698560 | C19ORF6 | 7.46E-03 | 17% | Chromosome 19 open reading frame 6, transcript variant 1. |
| **P-value RANK** | **PROBE_ID** | **GENE Symbol** | **Paired T-Test** | **% Change** | **DEFINITION** |
| 154 | ILMN_1806576 | LOC651137 | 7.48E-03 | -11% | PREDICTED: similar to T-complex protein 10A homolog, transcript variant 2. |
| 155 | ILMN_1821485 | HS.555706 | 7.49E-03 | -12% | NISC_gg04f02.x1 NCI_CGAP_Kid11 cDNA clone IMAGE:3253395 3. |
| 156 | ILMN_1747119 | FBXO46 | 7.58E-03 | -11% | PREDICTED: F-box protein 46, transcript variant 5. |
| 157 | ILMN_1684694 | ANK1 | 7.59E-03 | -8% | Ankyrin 1, erythrocytic, transcript variant 5. |
| 158 | ILMN_1898058 | HS.566297 | 7.59E-03 | -9% | zr42c11.s1 Soares_NhHMPu_S1 cDNA clone IMAGE:666068 3. |
| 159 | ILMN_1764207 | P15RS | 7.61E-03 | 15% | Hypothetical protein FLJ10656. |
| 160 | ILMN_1819989 | HS.574405 | 7.78E-03 | -15% | cDNA: FLJ21635 fis, clone COL08233, highly similar to AF131819 clone 24838 mRNA sequence |
| 161 | ILMN_1720850 | BAZ2B | 7.81E-03 | 10% | Bromodomain adjacent to zinc finger domain, 2B. |
| 162 | ILMN_1711089 | DNAJA5 | 7.92E-03 | 7% | DnaJ homology subfamily A member 5, transcript variant 1. |
| 163 | ILMN_1792682 | MCTP2 | 7.93E-03 | -9% | Multiple C2 domains, transmembrane 2. |
| 164 | ILMN_1775744 | MRPS16 | 7.95E-03 | -9% | Mitochondrial ribosomal protein S16, nuclear gene encoding mitochondrial protein. |
| 165 | ILMN_1723035 | **OLR1** | 8.09E-03 | -37% | **Oxidized low density lipoprotein (lectin-like) receptor 1.** |
| 166 | ILMN_1800855 | PPTC7 | 8.13E-03 | 12% | PTC7 protein phosphatase homolog (S. cerevisiae). |
| 167 | ILMN_1710394 | **CNTNAP5** | 8.14E-03 | -9% | **Contactin associated protein-like 5.** |
| 168 | ILMN_1759475 | TAS2R39 | 8.15E-03 | -9% | Taste receptor, type 2, member 39. |
| 169 | ILMN_1697412 | RHBG | 8.16E-03 | -13% | Rh family, B glycoprotein. |
| 170 | ILMN_1737819 | C7ORF16 | 8.16E-03 | -9% | Chromosome 7 open reading frame 16. |
| 171 | ILMN_1889178 | HS.157581 | 8.19E-03 | -8% | tf70b04.x5 NCI_CGAP_Brn23 cDNA clone IMAGE:2104591 3. |
| 172 | ILMN_1877890 | HS.551244 | 8.20E-03 | -8% | xs34e11.x1 NCI_CGAP_Kid11 cDNA clone IMAGE:2771564 3. |
| 173 | ILMN_1725510 | **DHCR24** | 8.21E-03 | -18% | **24-dehydrocholesterol reductase.** |
| 174 | ILMN_1789266 | CCDC25 | 8.28E-03 | 16% | Coiled-coil domain containing 25. |
| 175 | ILMN_1682487 | GPR133 | 8.29E-03 | -7% | G protein-coupled receptor 133. |
| 176 | ILMN_1678150 | OR6S1 | 8.31E-03 | -10% | Olfactory receptor, family 6, subfamily S, member 1. |
| 177 | ILMN_1814007 | LOC441488 | 8.43E-03 | -17% | PREDICTED: similar to Transcription factor Dp-1 (E2F dimerization partner 1) (DRTF1-polypeptide-1) (DRTF1), transcript variant 2. |
| 178 | ILMN_1891009 | HS.567064 | 8.53E-03 | -14% | xd96c08.x1 Soares_NFL_T_GBC_S1 cDNA clone IMAGE:2605454 3 similar to contains element MER40 repetitive element ;. |
| 179 | ILMN_1770454 | AGRN | 8.58E-03 | -9% | Agrin. |
| 180 | ILMN_1903750 | HS.544238 | 8.64E-03 | -8% | Human clone H8 Cri-du-chat critical region mRNA, partial sequence |
| 181 | ILMN_1741712 | MS4A4A | 8.67E-03 | -15% | Membrane-spanning 4-domains, subfamily A, member 4, transcript variant 1. |
| 182 | ILMN_1889426 | HS.554342 | 8.68E-03 | -8% | cong2.P5.a4 conorm cDNA 3. |
| 183 | ILMN_1658044 | LOC648814 | 8.70E-03 | 14% | PREDICTED: hypothetical protein LOC648814. |
| 184 | ILMN_1691418 | CDRT4 | 8.78E-03 | 20% | CMT1A duplicated region transcript 4. |
| 185 | ILMN_1721022 | SHC1 | 8.79E-03 | -6% | SHC (Src homology 2 domain containing) transforming protein 1, transcript variant 2. |
| 186 | ILMN_1687484 | ZFX | 8.84E-03 | 16% | Zinc finger protein, X-linked. |
| 187 | ILMN_1731168 | MBD1 | 8.84E-03 | -8% | Methyl-CpG binding domain protein 1, transcript variant 1. |
| 188 | ILMN_1812281 | ARG1 | 8.85E-03 | -11% | Arginase, liver. |
| 189 | ILMN_1777725 | LSM14B | 8.88E-03 | 9% | LSM14B, SCD6 homolog B (S. cerevisiae). |
| 190 | ILMN_1798032 | TRIM24 | 8.92E-03 | 15% | Tripartite motif-containing 24, transcript variant 2. |
| 191 | ILMN_1813400 | CBR4 | 8.96E-03 | 26% | Carbonyl reductase 4. |
| 192 | ILMN_1678775 | CLEC2D | 9.05E-03 | 19% | C-type lectin domain family 2, member D, transcript variant 1. |
| **P-value RANK** | **PROBE_ID** | **GENE Symbol** | **Paired T-Test** | **% Change** | **DEFINITION** |
| 193 | ILMN_1724825 | PCBP2 | 9.05E-03 | -8% | Poly(rC) binding protein 2, transcript variant 3. |
| 194 | ILMN_1888658 | HS.130260 | 9.11E-03 | 16% | qk02b10.x1 NCI_CGAP_Kid3 cDNA clone IMAGE:1867771 3. |
| 195 | ILMN_1734553 | LOC653234 | 9.27E-03 | 9% | PREDICTED: similar to centaurin, gamma-like family, member 1. |
| 196 | ILMN_1751214 | STARD3NL | 9.31E-03 | 15% | STARD3 N-terminal like. |
| 197 | ILMN_1707780 | C6ORF165 | 9.51E-03 | -11% | Chromosome 6 open reading frame 165, transcript variant 2. |
| 198 | ILMN_1670878 | YTHDC1 | 9.55E-03 | 12% | YTH domain containing 1, transcript variant 1. |
| 199 | ILMN_1749253 | TUBD1 | 9.58E-03 | 13% | Tubulin, delta 1. |
| 200 | ILMN_1755769 | MFSD8 | 9.73E-03 | 14% | Major facilitator superfamily domain containing 8. |
| 201 | ILMN_1789879 | **WDR35** | 9.87E-03 | 15% | **WD repeat domain 35, transcript variant 1.** |
| 202 | ILMN_1871496 | HS.213049 | 9.88E-03 | -13% | we11f11.x1 NCI_CGAP_Lu24 cDNA clone IMAGE:2340813 3. |
| 203 | ILMN_1687951 | LOC653480 | 9.98E-03 | -12% | PREDICTED: similar to transmembrane protein 23. |
| 204 | ILMN_1808305 | RTCD1 | 9.99E-03 | 15% | RNA terminal phosphate cyclase domain 1. |
