# Supplementary material for: Changes in Whole Blood Gene Expression in Obese Subjects with Type 2 Diabetes Following Bariatric Surgery: a Pilot Study
Source: PLoS One. 2011 Mar 10;6(3):e16729. doi: 10.1371/journal.pone.0016729 (PMC3053356; doi:10.1371/journal.pone.0016729)
Supplement: Table S3 — List represents significantly differentially expressed (paired t-test p-value<0.01) transcripts IDs whose changes in gene expression are correlated to changes in at least one of the following four clinical characteristics: weight, FPG, HbA1C or WBC, with an R2≥0.25. Values with R2≥0.25 are shown in bold. Gene symbols in blue are correlated to changes in weight, FPG, and HbA1c. (DOC) [file pone.0016729.s003.doc]

**Supplementary Table S3.** List represents significantly differentially expressed (paired t-test p-value < 0.01) transcripts IDs whose changes in gene expression are correlated to changes in at least one of the following four clinical characteristics: weight, FPG, HbA_1C_ or WBC, with an R^2^ > 0.25. Values with R^2^ ≥ 0.25 are shown in bold. Gene symbols in blue are correlated to changes in weight, FPG, and HbA_1c_.

| **PROBE_ID** | **GENE Symbol** | **ΔWeight** | | **ΔFPG** | | **ΔHbA_1c_** | | **ΔWBC** | |
| --- | --- | --- | --- | --- | --- | --- | --- | --- | --- |
|  |  | **R** | **R square** | **R** | **R square** | **R** | **R square** | **R** | **R square** |
| ILMN_1652604 | GGT1 | 0.12 | 0.01 | -0.24 | 0.06 | -0.35 | 0.12 | **0.63** | **0.39** |
| ILMN_1688580 | CAMP | **0.57** | **0.32** | **0.59** | **0.35** | 0.31 | 0.10 | **0.56** | **0.31** |
| ILMN_1682312 | CYBB | 0.50 | 0.25 | **0.65** | **0.42** | 0.37 | 0.14 | 0.13 | 0.02 |
| ILMN_1797682 | INSL3 | 0.14 | 0.02 | 0.34 | 0.12 | **0.52** | **0.27** | -0.02 | 0.00 |
| ILMN_1748384 | BOC | 0.06 | 0.00 | 0.02 | 0.00 | 0.34 | 0.12 | **-0.62** | **0.38** |
| ILMN_1679357 | DEFA1/DEFA3 | 0.36 | 0.13 | **0.56** | **0.31** | 0.46 | 0.21 | 0.38 | 0.14 |
| ILMN_1703324 | PDSS1 | **-0.80** | **0.63** | -0.32 | 0.11 | 0.00 | 0.00 | -0.05 | 0.00 |
| ILMN_1804631 | CNBP | **-0.53** | **0.29** | **-0.61** | **0.37** | -0.33 | 0.11 | -0.06 | 0.00 |
| ILMN_1833316 | HS.112596 | 0.38 | 0.15 | **0.56** | **0.31** | 0.22 | 0.05 | 0.09 | 0.01 |
| ILMN_1651544 | HDAC8 | -0.50 | 0.25 | **-0.65** | **0.43** | -0.48 | 0.23 | -0.47 | 0.22 |
| ILMN_1751393 | ZNF684 | -0.08 | 0.01 | **-0.50** | **0.25** | **-0.66** | **0.43** | 0.40 | 0.16 |
| ILMN_1769633 | CTSO | 0.21 | 0.04 | 0.37 | 0.13 | 0.36 | 0.13 | **0.76** | **0.58** |
| ILMN_1904282 | HS.484967 | -0.06 | 0.00 | -0.30 | 0.09 | **-0.66** | **0.44** | 0.07 | 0.00 |
| ILMN_1704973 | SARDH | 0.05 | 0.00 | 0.26 | 0.07 | **0.58** | **0.33** | -0.14 | 0.02 |
| ILMN_1652407 | ZMYND8 | -0.26 | 0.07 | **-0.59** | **0.35** | **-0.54** | **0.29** | **-0.77** | **0.59** |
| ILMN_1900247 | HS.147149 | -0.28 | 0.08 | 0.39 | 0.15 | **0.52** | **0.27** | 0.34 | 0.11 |
| ILMN_1651664 | SDHALP1 | **-0.51** | **0.26** | 0.01 | 0.00 | 0.20 | 0.04 | -0.25 | 0.06 |
| ILMN_1703858 | C7ORF24 | -0.34 | 0.12 | **-0.62** | **0.39** | **-0.61** | **0.37** | **-0.54** | **0.30** |
| ILMN_1768743 | FIP1L1 | -0.43 | 0.19 | **-0.54** | **0.29** | -0.34 | 0.11 | **-0.64** | **0.41** |
| ILMN_1684346 | TNFAIP8L1 | -0.22 | 0.05 | **-0.58** | **0.33** | **-0.53** | **0.28** | -0.44 | 0.20 |
| ILMN_1754772 | LOC650698 | **-0.51** | **0.26** | 0.05 | 0.00 | 0.37 | 0.13 | -0.14 | 0.02 |
| ILMN_1729175 | FBXO3 | **-0.56** | **0.31** | -0.42 | 0.18 | -0.32 | 0.10 | -0.25 | 0.06 |
| ILMN_1812578 | LOC645636 | -0.28 | 0.08 | -0.04 | 0.00 | 0.31 | 0.10 | **-0.76** | **0.58** |
| ILMN_1666024 | LOC642503 | 0.43 | 0.19 | **0.68** | **0.47** | 0.41 | 0.17 | 0.32 | 0.10 |
| ILMN_1699644 | MARCH3 | -0.01 | 0.00 | 0.46 | 0.21 | **0.60** | **0.36** | -0.27 | 0.08 |
| ILMN_1694483 | GPR50 | -0.01 | 0.00 | **-0.58** | **0.34** | **-0.74** | **0.55** | -0.14 | 0.02 |
| ILMN_1672108 | LOC388161 | 0.29 | 0.09 | **0.63** | **0.39** | 0.19 | 0.04 | 0.15 | 0.02 |
| ILMN_1676905 | **TIGD7** | **-0.52** | **0.27** | **-0.67** | **0.45** | **-0.64** | **0.41** | -0.20 | 0.04 |
| ILMN_1702858 | ADHFE1 | -0.20 | 0.04 | -0.33 | 0.11 | -0.12 | 0.01 | **-0.78** | **0.61** |
| ILMN_1656111 | MYLIP | -0.45 | 0.20 | **-0.56** | **0.32** | **-0.61** | **0.37** | 0.11 | 0.01 |
| ILMN_1689378 | CCRN4L | **0.57** | **0.33** | **0.66** | **0.43** | 0.24 | 0.06 | 0.36 | 0.13 |
| ILMN_1814009 | LOC283157 | -0.12 | 0.01 | 0.32 | 0.10 | 0.30 | 0.09 | **0.50** | **0.25** |
| ILMN_1911874 | HS.253554 | -0.10 | 0.01 | 0.12 | 0.02 | -0.08 | 0.01 | **0.63** | **0.40** |
| ILMN_1848556 | HS.125695 | -0.37 | 0.14 | -0.25 | 0.06 | -0.28 | 0.08 | **-0.53** | **0.28** |
| ILMN_1661940 | CAMTA1 | -0.36 | 0.13 | **-0.50** | **0.25** | **-0.52** | **0.27** | -0.44 | 0.20 |
| ILMN_1724634 | LOC645550 | 0.15 | 0.02 | 0.16 | 0.03 | 0.22 | 0.05 | **-0.56** | **0.31** |
| ILMN_1820198 | HS.576440 | -0.02 | 0.00 | 0.02 | 0.00 | 0.25 | 0.06 | **-0.76** | **0.58** |
| ILMN_1718354 | INTS7 | -0.34 | 0.12 | **-0.58** | **0.33** | **-0.67** | **0.45** | -0.03 | 0.00 |
| ILMN_1684929 | **TOPBP1** | **-0.55** | **0.30** | **-0.62** | **0.38** | **-0.58** | **0.34** | -0.11 | 0.01 |
| ILMN_1774971 | **FAM8A1** | **-0.77** | **0.60** | **-0.65** | **0.42** | **-0.50** | **0.25** | -0.12 | 0.01 |
| ILMN_1738246 | OR10A7 | -0.29 | 0.09 | -0.42 | 0.17 | **-0.52** | **0.27** | -0.18 | 0.03 |
| ILMN_1724240 | LBR | -0.46 | 0.21 | **-0.57** | **0.32** | -0.45 | 0.20 | 0.32 | 0.10 |
| ILMN_1718907 | **TSHZ1** | **-0.55** | **0.30** | **-0.74** | **0.55** | **-0.52** | **0.27** | -0.42 | 0.18 |
| ILMN_1662318 | CCDC59 | -0.41 | 0.16 | **-0.58** | **0.33** | -0.29 | 0.08 | -0.34 | 0.11 |
| ILMN_1779356 | TP53 | 0.29 | 0.09 | 0.07 | 0.01 | -0.14 | 0.02 | **0.81** | **0.66** |
| ILMN_1664718 | CYP51A1 | **-0.54** | **0.29** | -0.14 | 0.02 | 0.08 | 0.01 | **-0.60** | **0.36** |
| ILMN_1693853 | HHAT | -0.48 | 0.23 | **-0.79** | **0.62** | **-0.55** | **0.30** | **-0.64** | **0.41** |
| ILMN_1762713 | C19ORF59 | 0.18 | 0.03 | 0.39 | 0.15 | 0.32 | 0.11 | **0.80** | **0.64** |
| ILMN_1805271 | ZNF721 | -0.35 | 0.12 | **-0.58** | **0.33** | -0.30 | 0.09 | -0.28 | 0.08 |
| ILMN_1652409 | SPATA7 | **-0.58** | **0.33** | -0.44 | 0.19 | -0.32 | 0.10 | -0.27 | 0.07 |
| ILMN_1819255 | HS.428820 | 0.39 | 0.15 | **0.50** | **0.25** | 0.38 | 0.14 | 0.09 | 0.01 |
| ILMN_1751793 | PCNXL2 | 0.01 | 0.00 | 0.30 | 0.09 | **0.53** | **0.29** | -0.47 | 0.23 |
| ILMN_1764619 | **FLJ45244** | **0.68** | **0.46** | **0.77** | **0.60** | **0.73** | **0.53** | 0.33 | 0.11 |
| **PROBE_ID** | **GENE Symbol** | **ΔWeight** | | **ΔFPG** | | **ΔHbA_1c_** | | **ΔWBC** | |
|  |  | **R** | **R square** | **R** | **R square** | **R** | **R square** | **R** | **R square** |
| ILMN_1698560 | C19ORF6 | **0.70** | **0.49** | 0.35 | 0.13 | 0.22 | 0.05 | 0.15 | 0.02 |
| ILMN_1806576 | LOC651137 | -0.18 | 0.03 | -0.17 | 0.03 | 0.30 | 0.09 | **-0.50** | **0.25** |
| ILMN_1821485 | HS.555706 | -0.03 | 0.00 | -0.28 | 0.08 | **-0.53** | **0.28** | 0.34 | 0.12 |
| ILMN_1747119 | FBXO46 | **0.53** | **0.28** | 0.21 | 0.04 | -0.13 | 0.02 | 0.12 | 0.02 |
| ILMN_1898058 | HS.566297 | -0.19 | 0.04 | 0.17 | 0.03 | 0.35 | 0.12 | **-0.58** | **0.34** |
| ILMN_1819989 | HS.574405 | **0.56** | **0.32** | 0.44 | 0.19 | 0.17 | 0.03 | 0.09 | 0.01 |
| ILMN_1711089 | DNAJA5 | -0.22 | 0.05 | **-0.59** | **0.35** | -0.34 | 0.11 | 0.26 | 0.07 |
| ILMN_1775744 | MRPS16 | 0.16 | 0.02 | -0.07 | 0.00 | -0.03 | 0.00 | **-0.62** | **0.38** |
| ILMN_1710394 | CNTNAP5 | 0.05 | 0.00 | 0.26 | 0.07 | **0.52** | **0.27** | -0.39 | 0.15 |
| ILMN_1889178 | HS.157581 | -0.12 | 0.01 | -0.28 | 0.08 | -0.20 | 0.04 | **0.51** | **0.26** |
| ILMN_1725510 | **DHCR24** | **0.62** | **0.39** | **0.62** | **0.39** | **0.59** | **0.35** | 0.31 | 0.09 |
| ILMN_1889426 | HS.554342 | **0.76** | **0.58** | **0.62** | **0.39** | 0.20 | 0.04 | **0.59** | **0.35** |
| ILMN_1721022 | SHC1 | 0.14 | 0.02 | -0.23 | 0.05 | **-0.51** | **0.27** | -0.35 | 0.12 |
| ILMN_1687484 | ZFX | -0.42 | 0.17 | **-0.54** | **0.29** | **-0.53** | **0.28** | -0.21 | 0.05 |
| ILMN_1812281 | ARG1 | 0.03 | 0.00 | 0.26 | 0.07 | 0.17 | 0.03 | **0.69** | **0.47** |
| ILMN_1777725 | LSM14B | -0.21 | 0.04 | -0.17 | 0.03 | **-0.53** | **0.28** | 0.06 | 0.00 |
| ILMN_1798032 | TRIM24 | -0.32 | 0.10 | -0.39 | 0.15 | **-0.57** | **0.32** | 0.12 | 0.01 |
| ILMN_1813400 | CBR4 | -0.47 | 0.22 | **-0.66** | **0.44** | **-0.74** | **0.54** | -0.23 | 0.05 |
| ILMN_1751214 | STARD3NL | -0.11 | 0.01 | -0.38 | 0.14 | **-0.50** | **0.25** | 0.25 | 0.06 |
| ILMN_1707780 | C6ORF165 | -0.10 | 0.01 | 0.23 | 0.05 | **0.62** | **0.38** | -0.18 | 0.03 |
| ILMN_1789879 | **WDR35** | **-0.53** | **0.28** | **-0.62** | **0.39** | **-0.75** | **0.56** | -0.25 | 0.06 |
| ILMN_1871496 | HS.213049 | 0.35 | 0.12 | 0.44 | 0.19 | 0.46 | 0.21 | **0.55** | **0.30** |
