# Supplementary material for: Changes in Whole Blood Gene Expression in Obese Subjects with Type 2 Diabetes Following Bariatric Surgery: a Pilot Study
Source: PLoS One. 2011 Mar 10;6(3):e16729. doi: 10.1371/journal.pone.0016729 (PMC3053356; doi:10.1371/journal.pone.0016729)
Supplement: Table S4 — Table shows the gene ontology (GO) terms and pathways identified by gene ontology and functional analyses (DAVID) that were overrepresented (P-value<0.01) in our list of 204 significantly differentially expressed transcripts after bariatric surgery. GO terms discussed in the manuscripts are shown in bold. (DOC) [file pone.0016729.s004.doc]

**Supplementary Table S4:** Table shows the gene ontology (GO) terms and pathways identified by gene ontology and functional analyses (DAVID) that were overrepresented (P-value < 0.01) in our list of 204 significantly differentially expressed transcripts after bariatric surgery. GO terms discussed in the manuscripts are shown in bold.

| **20 Transcripts were found to be best correlated with weight changes, only 18 were recognized and included in gene ontology analysis.** | | | |  |
| --- | --- | --- | --- | --- |
|  |  |  |  |  |
| **Term Resource and ID** | **Term Name** | **Count** | **% of genes involved/total genes** | **P-Value** |
| GO:0006695 | **Cholesterol biosynthetic process** | 3 | 17.65% | **1.23E-04** |
| GO:0016126 | **Sterol biosynthetic process** | 3 | 17.65% | **2.12E-04** |
| GO:0008610 | Lipid biosynthetic process | 4 | 23.53% | 6.41E-04 |
| GO:0008203 | Cholesterol metabolic process | 3 | 17.65% | 1.06E-03 |
| GO:0006694 | Steroid biosynthetic process | 3 | 17.65% | 1.29E-03 |
| GO:0016125 | Sterol metabolic process | 3 | 17.65% | 1.39E-03 |
| KEGG_PATHWAY | Biosynthesis of steroids | 2 | 11.76% | 5.70E-03 |
| GO:0008202 | Steroid metabolic process | 3 | 17.65% | 6.22E-03 |
| GO:0044255 | Cellular lipid metabolic process | 4 | 23.53% | 6.46E-03 |
|  |  |  |  |  |
| **35 Transcripts were found to be best correlated with fasting plasma glucose changes, only 32 were recognized and included in gene ontology analysis.** | | | | |
|  |  |  |  |  |
| **Term Resource and ID** | **Term Name** | **Count** | **% of genes involved/total genes** | **P-Value** |
| GO:0006139 | Nucleobase, nucleoside, nucleotide and nucleic acid metabolic process | 14 | 43.75% | 6.47E-04 |
| GO:0016070 | RNA metabolic process | 12 | 37.50% | 8.27E-04 |
| GO:0005634 | Nucleus | 15 | 46.88% | 9.00E-04 |
| GO:0010467 | Gene expression | 13 | 40.62% | 1.14E-03 |
| GO:0006350 | Transcription | 11 | 34.38% | 1.46E-03 |
| GO:0043283 | Biopolymer metabolic process | 15 | 46.88% | 2.98E-03 |
| GO:0006351 | Transcription, DNA-dependent | 10 | 31.25% | 3.45E-03 |
| GO:0032774 | RNA biosynthetic process | 10 | 31.25% | 3.47E-03 |
| GO:0003677 | DNA binding | 9 | 28.12% | 4.64E-03 |
| GO:0045449 | Regulation of transcription | 10 | 31.25% | 4.67E-03 |
| GO:0003676 | Nucleic acid binding | 11 | 34.38% | 4.93E-03 |
| GO:0019219 | Regulation of nucleobase, nucleoside, nucleotide and nucleic acid metabolic process | 10 | 31.25% | 5.47E-03 |
| SP_PIR_KEYWORDS | Nucleus | 12 | 37.50% | 5.76E-03 |
| GO:0010468 | Regulation of gene expression | 10 | 31.25% | 7.06E-03 |
| SP_PIR_KEYWORDS | DNA-binding | 8 | 25.00% | 7.15E-03 |
| SP_PIR_KEYWORDS | Transcription regulation | 8 | 25.00% | 7.28E-03 |
| SP_PIR_KEYWORDS | Transcription | 8 | 25.00% | 7.96E-03 |
| GO:0031323 | Regulation of cellular metabolic process | 10 | 31.25% | 9.25E-03 |
